# Effects of a Diet Based on Foods from Symbiotic Agriculture on the Gut Microbiota of Subjects at Risk for Metabolic Syndrome

**DOI:** 10.3390/nu13062081

**Published:** 2021-06-17

**Authors:** Silvia Turroni, Elisabetta Petracci, Valeria Edefonti, Anna M. Giudetti, Federica D’Amico, Lisa Paganelli, Giusto Giovannetti, Laura Del Coco, Francesco P. Fanizzi, Simone Rampelli, Debora Guerra, Claudia Rengucci, Jenny Bulgarelli, Marcella Tazzari, Nicoletta Pellegrini, Monica Ferraroni, Oriana Nanni, Patrizia Serra

**Affiliations:** 1 Department of Pharmacy and Biotechnology, University of Bologna, via Belmeloro, 6, 40126 Bologna, Italy; silvia.turroni@unibo.it (S.T.); federica.damico8@unibo.it (F.D.); simone.rampelli@unibo.it (S.R.); 2 Biostatistics and Clinical Trial Unit, Istituto Romagnolo per lo Studio dei Tumori “Dino Amadori”—IRST S.r.l., IRCCS, via P. Maroncelli, 40, 47014 Meldola, Italy; elisabetta.petracci@irst.emr.it (E.P.); oriana.nanni@irst.emr.it (O.N.); patrizia.serra@irst.emr.it (P.S.); 3 Branch of Medical Statistics, Biometry, and Epidemiology “G.A. Maccacaro”, Department of Clinical Sciences and Community Health, Università degli Studi di Milano, via A. Vanzetti, 5, 20133 Milano, Italy; monica.ferraroni@unimi.it; 4 Department of Biological and Environmental Sciences and Technologies, University of Salento, via Province of Lecce—Monteroni, 73100 Lecce, Italy; anna.giudetti@unisalento.it (A.M.G.); laura.delcoco@unisalento.it (L.D.C.); fp.fanizzi@unisalento.it (F.P.F.); 5 Department of Medical and Surgical Sciences, University of Bologna, via Massarenti, 9, 40138 Bologna, Italy; 6 Donna Impresa Coldiretti Forlì-Cesena e Rimini, via E. Forlanini, 11, 47121 Forli, Italy; l.paganelli@hotmail.com; 7 Centro Colture Sperimentali, CCS Aosta S.r.l., Frazione Olleyes 9, 11020 Quart, Italy; giustogiovannetti@hotmail.com; 8 Department of Medical Oncology, Istituto Romagnolo per lo Studio dei Tumori “Dino Amadori”—IRST S.r.l., IRCCS, via P. Maroncelli, 40, 47014 Meldola, Italy; debora.guerra@irst.emr.it; 9 Bioscience Laboratory, Istituto Romagnolo per lo Studio dei Tumori “Dino Amadori”—IRST S.r.l., IRCCS, via P. Maroncelli, 40, 47014 Meldola, Italy; claudia.rengucci@irst.emr.it; 10 Immunotherapy, Cell Therapy and Biobank Unit, Istituto Romagnolo per lo Studio dei Tumori “Dino Amadori”—IRST S.r.l., IRCCS, via P. Maroncelli, 40, 47014 Meldola, Italy; jenny.bulgarelli@irst.emr.it (J.B.); marcella.tazzari@irst.emr.it (M.T.); 11 Department of Agricultural, Food, Environmental and Animal Sciences, Università di Udine, via delle Scienze 206, 33100 Udine, Italy; nicoletta.pellegrini@uniud.it

**Keywords:** adult volunteers, dietary intervention, dietary patterns, gut microbiota, pilot study, symbiotic agriculture, metabolic dysfunction, metabolic syndrome, metabolome

## Abstract

Diet is a major driver of gut microbiota variation and plays a role in metabolic disorders, including metabolic syndrome (MS). Mycorrhized foods from symbiotic agriculture (SA) exhibit improved nutritional properties, but potential benefits have never been investigated in humans. We conducted a pilot interventional study on 60 adults with ≥ 1 risk factors for MS, of whom 33 consumed SA-derived fresh foods and 27 received probiotics over 30 days, with a 15-day follow-up. Stool, urine and blood were collected over time to explore changes in gut microbiota, metabolome, and biochemical, inflammatory and immunologic parameters; previous dietary habits were investigated through a validated food-frequency questionnaire. The baseline microbiota showed alterations typical of metabolic disorders, mainly an increase in *Coriobacteriaceae* and a decrease in health-associated taxa, which were partly reversed after the SA-based diet. Improvements were observed in metabolome, MS presence (two out of six subjects no longer had MS) or components. Changes were more pronounced with less healthy baseline diets. Probiotics had a marginal, not entirely favorable, effect, although one out of three subjects no longer suffered from MS. These findings suggest that improved dietary patterns can modulate the host microbiota and metabolome, counteracting the risk of developing MS.

## 1. Introduction

Metabolic syndrome (MS) is characterized by several metabolic abnormalities, including abdominal obesity, elevated values of triglycerides, blood pressure, or fasting glucose, or reduced high-density lipoprotein (HDL) cholesterol [1]. This pathological condition has been increasing over recent years, mainly due to changes in lifestyle and unbalanced diets, with a prevalence of 10–40% in the European population, depending on age and gender [2,3]. Subjects with untreated MS can easily develop cardiovascular and cerebrovascular disease, with an increased risk of mortality [4]. Moreover, several epidemiological and clinical studies support the hypothesis that MS may also be an important etiologic factor for the development and progression of certain types of cancer and for overall cancer mortality [5,6,7,8,9].

An increasing number of studies have shown that the gut microbiota (i.e., the vast and diverse set of microorganisms that populate our intestine) may play a role in the pathogenesis and progression of MS [10,11,12,13,14]. In particular, the studies are consistent in highlighting a dysbiotic (i.e., unbalanced) profile, comparable to that observed in other metabolic disorders, characterized by: (i) reduced diversity (a well-recognized hallmark of healthy gut and overall health); (ii) reduced proportions of beneficial commensals, mostly short-chain fatty acid (SCFA) producers; and (iii) increased amounts of opportunistic pathogens or pathobionts, including proteobacteria and other taxa whose pathogenic potential has only recently been revealed, for example *Coriobacteriaceae*. This layout could contribute in various ways to MS, e.g., by affecting satiety, favoring fat storage, altering intestinal cholesterol absorption, reducing hepatic glycogenesis and increasing triglyceride synthesis, exerting prothrombotic and hypertensive effects and, not least, disrupting the integrity of the epithelial barrier, thus consolidating a chronic low-grade inflammatory state [15,16,17,18,19]. It is, therefore, not surprising that the gut microbiota has been proposed as a target in interventions aimed at mitigating the risk of MS. In particular, given its sensitivity to variations in the amount of food and especially to the composition of the diet itself [20,21], dietary interventions for microbiota modulation, including the supplementation of pre- and probiotics, have been and still are the subject of numerous and recent studies [2,15,22].

Symbiotic agriculture (SA) is an agricultural production process aimed at restoring, safeguarding and employing in agro-ecosystems the natural symbiosis present between soil microorganisms (mainly fungi and bacteria) and the plant systems of cultivated species. The objectives that animate this new vision of agriculture are to: (i) increase the sustainability of agricultural practices by favoring the mechanisms to restore the biological fertility of soils and bio-sequestration of carbon, increasing the efficiency of crop fertilization interventions and reducing greenhouse gas emissions from the soil; (ii) increase the resistance of crops to adversity; (iii) increase the yield of crops (over-yielding) and plantations with off-land and under-earth luxuration by re-functionalizing N-organic; (iv) produce food and feed with greater shelf-life and greater transferable antioxidant and secondary metabolites relevant to human health; and (v) increase and improve the nutritional properties and the natural content of vitamins and metabolites produced exclusively by associated microorganisms, such as cobalamin (B12) and menaquinone (K2) [23,24,25]. In particular, SA systems make extensive use of mycorrhizal fungi and bacteria as ecologically and economically relevant fertilizers, which contribute to ecosystem functioning and crop productivity. The ultimate impact of mycorrhized farming on the nutritional and nutraceutical value of derived foods, such as fruits, vegetables, legumes and cereals, has recently been proven, especially in terms of antioxidant capacity, phenolic content and secondary metabolites levels [24,25,26]. However, as far as we know, to date, no SA-derived foods have been tested in a dietary intervention on humans.

To investigate whether a diet based on SA-derived products could impact the gut microbiota of subjects at risk for MS, we designed a pilot interventional study where subjects with at least one predisposing MS factor were provided with fresh food products from local certified organic SA production for 30 days. In parallel, a second group of subjects with similar characteristics followed their usual diet, except for probiotic supplementation. After a baseline interview, including an assessment of usual dietary habits, subjects were asked to collect fecal samples at baseline and during intervention until the end of follow-up for microbiota profiling.

In addition, through a comprehensive characterization of the enrolled subjects, the study aimed to provide preliminary evidence for other potential effects of a SA-based intervention, such as those on anthropometric and biochemical factors, urine metabolome profile, and the inflammatory and immunological status of the included subjects. All these aspects were integrated and analyzed with respect to the baseline dietary profile of study participants.

## 2. Materials and Methods

### 2.1. Study Setting and Participants

This pilot intervention study was promoted and conducted by the Istituto Romagnolo per lo Studio dei Tumori “Dino Amadori” (IRST) between October 2018 and September 2019 in the catchment area of Romagna, Italy. Subjects aged 18–65 years and with at least one of the following conditions were eligible for enrollment: abdominal obesity, hypertension, dyslipidemia, impaired fasting glucose or insulin resistance (for the formal definition of MS see Table 1 of Alberti et al., 2009 [27]). Subjects with severe or uncontrolled conditions or under treatment with antibiotics or following a specific diet regimen, such as vegan or celiac individuals, were excluded from the study.

To promote and facilitate study recruitment, an illustrative brochure was distributed in the IRST area. Moreover, a press conference was organized, and the study was promoted in local newspapers. In all cases, information on the study and reference staff was provided.

The protocol was approved by the CEROM Ethical Committee (Study ID: IRST B086 L4P1755, Ethical approval ID: 6759/2018). All participants provided written informed consent or assent.

### 2.2. Study Intervention

The study was designed to enroll two groups of subjects: those receiving fresh foods from organic symbiotic crops with mycorrhizae (SA-group) and those integrating their habitual diet with receiving probiotics with sachet formulation (PROB-group). Subjects from both groups were asked not to change their usual diet during the study period. The dietary intervention lasted 30 days. For a preliminary evaluation of the durability of potential changes in the gut microbiota after intervention, a follow-up of 15 days was planned. Although comparison between the two groups was not an objective of the present study, subjects were randomly allocated to one of the two groups using the nQuery Advisor^®^, Version 7.0 (Statsols, Statistical Solutions Limited, Cork, Ireland) mixed block non-stratified randomization procedure.

Subjects in the SA-group substituted their habitual foods with fresh, baked or steamed products chosen from a ~130-item menu-like list based on seasonal availability, including: fruit and vegetables in season (e.g., apples, pears, kiwis, beetroot, broccoli, cabbage, carrots, cauliflower, celery chickpeas, beans, fennel, salad, lentils, potatoes, spinach, tomatoes, and pumpkin); whole wheat or spelt-based products (bread, pasta, bakery products, including focaccia, breadsticks, cakes, and biscuits, as well as flour to be directly used in recipes); dairy products (e.g., milk, cheese, and yoghurt) from cattle, sheep, and goats; different cuts of poultry and red meat; extra virgin olive oil; tomato sauce and pesto; jam and fruit juices. All the products were derived from local certified organic SA production and prepared by farmers; therefore, they were free from colorants, preservatives or other additives normally present in preserved foods; they were delivered twice a week directly to each volunteer’s home. The diet also included the use of aromatic herbs.

The probiotics provided to the subjects in the PROB-group were manufactured by Probiotical S.p.A., Novara (Italy). Each sachet included LF08 (*Lactobacillus fermentum*), LP09 (*Lactobacillus plantarum*), and LS01 (*Lactobacillus salivarius*) at 3.33 billion CFUs each. Maltodextrin was used as the excipient. This mixture was chosen based on previous studies that showed a beneficial effect of these *Lactobacillus* species on markers of MS [28,29,30]. Each individual in PROB-group was asked to take one 2.5-g sachet every day.

### 2.3. Collection of Participants’ Information and Samples

At baseline, a nutritional visit by trained personnel allowed to collect demographic and anamnestic information and to measure anthropometry. Dietary habits over the past year were also assessed through the self-administration of the European Prospective Investigation into Cancer and Nutrition (EPIC) food-frequency questionnaire (FFQ) [31,32]. No nutritional counselling was provided during the visit. In addition, a blood sample for the determination of biochemical parameters and cytokine levels, and one sample of stool and urine for the characterization of the microbiota and metabolome, respectively, were obtained from all subjects.

**Figure 1:**
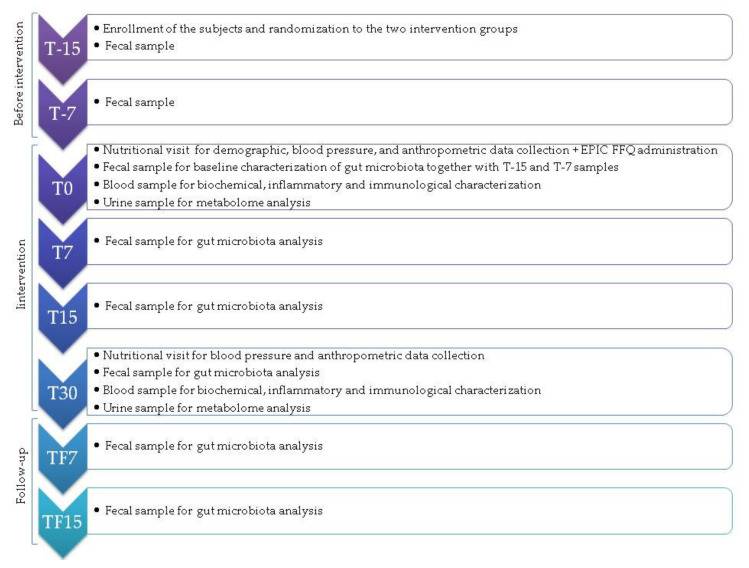
Study timeline. Italy, 2018–2019. The time points are grouped as follows: (i) T-15 and T-7: 15 and 7 days before the intervention (Before intervention); (ii) T0: start of the intervention; T7, T15 and T30: 7, 15 and 30 days from the beginning of the intervention (Intervention); and (iii) TF7 and TF15: 7 and 15 days after the end of the intervention (Follow-up). The validated semi-quantitative European Prospective Investigation into Cancer and Nutrition (EPIC) Food Frequency Questionnaire (FFQ) was administered to collect information on consumption frequency of food items.

The biochemical parameters were immediately assessed, whereas serum was separated by centrifuging the blood samples and stored at −80 °C until use. The serum levels of the human inflammatory cytokines IFN-γ, IL-6, IL-10, IL-17A and TNF-α were measured by a multiplexed bead-based immunoassay (Flex set Cytometric Bead Array (CBA), BD Bioscience, San Jose, CA, USA). Samples were acquired with the FACSCanto flow cytometer (BD Bioscience) and the data were analyzed by Diva software and CBA software (BD Bioscience). Fecal samples were collected in sterile containers and stored at −80 °C at IRST Bioscience Laboratory before being shipped on dry ice to the Microbiology Laboratory at the Department of Pharmacy and Biotechnology, University of Bologna (Bologna, Italy) for gut microbiota analysis. Urine samples were collected in sterile containers on the same day of blood collection and stored at −80 °C at IRST Bioscience Laboratory before being shipped on dry ice to the General and Inorganic Chemistry Laboratory at the Department of Biological and Environmental Sciences and Technologies, University of Salento (Lecce, Italy). Anthropometric information and biological samples were collected at multiple time points before and during intervention as well as in the follow-up (see Figure 1 for more details).

The study lasted 30 days, with a 15-day follow-up (TF). Fecal samples were collected weekly in the two-week run-in period for a more reliable depiction of the basal microbiota configuration. Biological sample collection and anthropometric, biochemical and immunological measurements were performed at multiple time points as shown. Dietary habits were assessed at baseline using a validated food frequency questionnaire (see Figure 1 for more details).

### 2.4. Dietary Habits at Baseline

The current study collected information on consumption frequency of food items as derived at baseline from the 188-item validated semi-quantitative EPIC FFQ [33], designed to capture local dietary habits for the Varese, Turin, and Florence centers [32]. Estimates of daily intakes of energy, minerals, macro- and micro-nutrients (altogether indicated as “nutrients”, hereafter) were derived by linking the food items with the Italian Food Composition Tables [34] through a dedicated software package [31].

### 2.5. Gut Microbiota Analysis through Illumina Sequencing

Microbial DNA was extracted from fecal samples using the repeated bead-beating plus column method [35] with a few modifications [36]. For the baseline, the feces of three replicates (collected weekly, i.e., at T-15, T-7, and T0, see Figure 1) were pooled together. A parallel sensitivity analysis explored the baseline variation at the separate T-15, T-7, and T0 time-points, with an additional focus on genera that changed significantly during SA-based diet. Feces processing was performed as described below. Briefly, approximately 250 mg of each sample was suspended in 1 mL of lysis buffer with four 3-mm glass beads and 0.5 g of 0.1-mm zirconia beads (BioSpec Products, Bartlesville, OK, USA), and bead-beaten in a FastPrep homogenizer (MP Biomedicals, Irvine, CA, USA) at 5.5 movements/s for 1 min three times. The samples were then incubated for 15 min at 95 °C and centrifuged at 13,000 rpm for 5 min. The supernatants were added with 260 μL of 10 M ammonium acetate, and incubated for 30 min with isopropanol (one volume). After washing with 70% ethanol, the nucleic acid pellet was suspended in 100 µL of TE buffer. RNA was removed by 15-min incubation with 2 μL of DNase-free RNase (10 mg/mL) at 37 °C. For the subsequent DNA purification steps, the DNeasy Blood and Tissue Kit (QIAGEN, Hilden, Germany) was used. DNA was assessed for concentration and quality using the NanoDrop ND-1000 spectrophotometer (NanoDrop Technologies, Wilmington, DE, USA).

The V3–V4 hypervariable region of the 16S rRNA gene was amplified using primers 341F and 785R [37], including overhang adapter sequences for Illumina sequencing. For amplification, KAPA HiFi HotStart ReadyMix (Roche, Basel, Switzerland) was used with the following thermal cycle: 95 °C for 3 min, 25 cycles of 95 °C for 30 s, 55 °C for 30 s, and 72 °C for 30 s, and 72 °C for 5 min. Amplicons were purified using magnetic beads (Agencourt AMPure XP, Beckman Coulter, Brea, CA, USA). A limited-cycle PCR was used to add Illumina sequencing adapters and barcodes. After another purification step, samples were pooled at equimolar concentration of 4 nM, denatured and diluted to 5 pM. The final library was sequenced on an Illumina MiSeq platform following a 2 × 250 bp paired-end protocol per manufacturer’s instructions (Illumina, San Diego, CA, USA). Raw sequencing reads were deposited in the National Center for Biotechnology Information Sequence Read Archive (Bioproject ID PRJNA726866).

For sequence processing, PANDASeq [38] and QIIME 2 [39] were used. Reads were filtered for length and quality, and subsequently binned into amplicon sequence variants (ASVs) using DADA2 [40]. The VSEARCH algorithm [41] and the Greengenes database (May 2013 release) were used for taxonomic assignment. Chimeras were discarded during the analysis. Different alpha diversity metrics, such as the inverse Simpson index, Faith’s Phylogenetic Diversity (PD whole tree) and the number of observed ASVs, were used. For beta diversity, weighted and unweighted UniFrac distances and Bray-Curtis dissimilarity were used to construct Principal Coordinates Analysis (PCoA) graphs. Publicly available sequences of the gut microbiota from age- and sex-matched healthy Italians were downloaded and processed as above. Specifically, we recovered sequences from De Filippis et al. (45 Italian adults; NCBI SRA SRP042234) [42], Schnorr et al. (2 Italian adults; MG-RAST mgp12183) [43] and Biagi et al. (13 Italian adults; MG-RAST mgp17761) [44].

### 2.6. Urine Metabolomics by Nuclear Magnetic Resonance Spectroscopy

For the Nuclear Magnetic Resonance (NMR) Spectroscopy analysis, 540 μL of urine, thawed at room temperature and mixed, was added to 60 μL of saline buffer solution (KH_2_PO_4_, in 100% D_2_O containing 0.03% *w*/*w* TSP as chemical shift reference and 2 mM sodium azide, pH 7.4), and transferred into a 5mm NMR tube. ^1^H-NMR spectra were acquired using a Bruker Avance III 600 Ascend NMR spectrometer (Bruker, Milan, Italy), operating at 600.13 MHz for 1H observation, equipped with a TCI cryoprobe (Triple Resonance inverse Cryoprobe) incorporating a z-axis gradient coil and automatic tuning-matching (ATM). Samples were loaded on a Bruker Automatic Sample Changer, interfaced with the IconNMR software (Bruker, Milan, Italy), and analyzed in automatic mode, setting a time delay of 5 min between sample injection and pre-acquisition calibrations for complete temperature equilibration (300 K). Measurements were repeated once in random order after the completion of the first entire set. For each sample, a standard 1D ^1^H-NMR (ZGCPPR Bruker standard pulse sequence) spectrum, with pre-saturation and composite pulse for selection, was recorded, with 64 transients, 16 dummy scans, 5s relaxation delay, size of FID (free induction decay) of 64 K data points, spectral width of 12,019.230 Hz (20.0276 ppm), acquisition time of 2.73 s and saturation of the solvent signal during the relaxation delay. The resulting FIDs were multiplied by an exponential weighting function equivalent to a line broadening of 0.3 Hz prior to Fourier transformation, automated phasing and baseline correction. Molecular constituent identification was performed by analysis of several spectroscopic NMR data. The compounds were identified by correspondence with literature data [45], according to their chemical shifts, multiplicity and homonuclear and heteronuclear coupling, exhibited in the 1D and 2D NMR spectra. In particular, ^1^H-^1^H J-resolved, ^1^H-^1^H COSY Correlation Spectroscopy, ^1^H-^13^C HSQC Heteronuclear Single Quantum Correlation, ^1^H-^13^C HMBC, Heteronuclear Multiple Bond Correlation NMR experiments and a freely available electronic database containing detailed information about metabolites were used (see https://hmdb.ca/, last accessed on 17 May 2021, and reference [46]). NMR data were processed using TopSpin 3.6.1 and Analysis of Mixture, Amix 3.9.13 (Bruker, Biospin, Milan, Italy), for both simultaneous visual inspection and the successive bucketing process.

### 2.7. Statistical Analyses

Participant characteristics were summarized by means of descriptive statistics such as median, minimum and maximum values or interquartile range (IQR) for continuous variables, and frequencies and percentages for categorical ones. Student’s *t*-test or the Mann Whitney U test and the Chi-square or the Fisher’s exact test, as appropriate, were used to compare baseline characteristics (i.e., demographic, anthropometric, biochemical parameters, cytokines, and actual adherence to a Mediterranean-style diet) between SA-group and PROB-group. As cytokines presented with highly skewed distributions, some preliminary data transformations were attempted within the Box-Cox family. However, none of them significantly improved the original skewness, given the presence of several zeros, and therefore the analyses were performed on the untransformed data. To compare the above-mentioned data over time (at baseline, T0, and after intervention, T30), the paired *t*-test, the Wilcoxon signed rank test, or the McNemar test was used, as appropriate.

Adherence to a Mediterranean-style diet was assessed by the calculation of the Italian Mediterranean Index (IMI), which was designed to specifically target dietary habits of the Italian population [47], measured by the EPIC FFQ as in our study population. Briefly, this score considered intakes of 11 items, including 6 typical Mediterranean foods (pasta; typical Mediterranean vegetables such as raw tomatoes, leafy vegetables, onion, and garlic, salad, and fruiting vegetables; fruit; legumes; olive oil; and fish), 4 non-Mediterranean foods (soft drinks, butter, red meat, and potatoes) and alcohol. Subjects received 1 point if consumption of typical Mediterranean foods was in the 3rd tertile of the distribution, and 0 points otherwise; when consumption of non-Mediterranean foods was in the first tertile of the distribution, the study participant received 1 point and 0 points otherwise. Ethanol intakes up to 12 g d^−1^ received 1 point, while abstainers and persons who consumed <12 g d^−1^ scored 0. Possible scores ranged from 0 to 11. Details on component definition and standard portions for optimal scoring were provided in [47]. The final index was then divided based on the final categories provided in [47] to improve comparability. Comparisons across the three IMI categories were conducted by referring to the Kruskal Wallis test and the Chi-square or the Fisher’s exact test, as appropriate.

An exploratory factor analysis (EFA) was carried out on a selected list of 27 nutrients to summarize overall dietary behavior at baseline in terms of a smaller number of underlying unobservable and randomly varying factors, which can be interpreted as dietary patterns (DPs) derived from EFA (EFA-based DPs). After factorability checks on the nutrient-based correlation matrix (visual inspection, Bartlett’s test of sphericity, overall and individual measures of sampling adequacy), the main analysis was based on: (i) principal component method; (ii) eigenvalue >1 and scree-plot criteria, to choose how many factors to retain; (iii) varimax rotation, to make factor naming easier; and (iv) 0.63 cut-off criterion for factor labeling. To quantify the adherence of each subject’s diet to each EFA-based DP, we estimated the factor scores for each subject and DP, following the weighted least squares method. We further calculated the Pearson correlation coefficients between the EFA-based DP scores and the daily amount of 37 selected food groups and condiments, derived from the original food items on the same subjects (see for example [48,49] for a more detailed description of the methodology). A cluster analysis (CLU) was carried out on the EFA-based DP scores to further classify subjects according to one (and only one) indicator of similarity in dietary habits among subjects at baseline (CLU-based DP or dietary cluster) [50]. We adopted the Partitioning Around Medoids (PAM) CLU algorithm: as compared to k-means, the PAM algorithm is less sensitive to outliers, and it is integrated with the average silhouette method to choose the optimal number of clusters [50]. Either Euclidean or Manhattan distances were considered, with similar results; the Euclidean distance was selected for the final analysis. The results of the average silhouette method were integrated with model parsimony and cluster interpretation, for the final decision on the optimal number of clusters to retain. Cluster labeling was qualitative and relied on the position of each cluster center within the ranges of the factor scores used as input data. A sensitivity analysis was also conducted considering other clustering methods, including hierarchical clustering and Gaussian mixture models.

For microbiota analysis, the significance of separation in PCoA of beta diversity between study subjects and age- and sex-matched healthy Italians, as well as within each intervention group over time, was tested by a permutation test with pseudo-F ratio (function “adonis” in the R vegan package). To assess differences in alpha diversity and microbiota composition among groups, Kruskal–Wallis or Friedman tests followed by post hoc Wilcoxon tests (paired or unpaired as needed) were performed. Kendall rank correlation test was used to assess the associations between genus-level relative abundances and anthropometric, biochemical, immunological and metabolomic variables. Only statistically significant correlations with absolute Kendall’s tau ≥0.2 were considered. As for the integration with dietary information, differences in beta diversity and composition at various phylogenetic levels were evaluated across the different clusters defined at baseline. In addition, the food groups and condiments most contributing to the ordination space were identified using the function “envfit” of the R vegan package. When appropriate, *p*-values were corrected for multiple comparisons using the Benjamini–Hochberg or false discovery rate (FDR) method. An FDR-adjusted *p*-value ≤ 0.05 was considered as statistically significant. A *p*-value between 0.05 and 0.1 was considered a tendency.

To investigate, within a unified framework, whether changes over time at genus level were associated with any temporal improvement in the components of MS, the nonparametric rank-based longitudinal methodology proposed by Noguchi et al., 2012 [51] was applied. This method is robust to outliers, heavily skewed data, and has competitive performance for small sample sizes compared to its parametric counterpart. The factorial design chosen considered one whole-plot factor, stratifying subjects in independent groups, and one sub-plot factor, a time variable for the six repeated measures (T0, T7, T15, T30, TF7, and TF15). All analyses were performed for each genus separately and for each intervention group. Two alternative versions of the whole-plot factor were proposed. The former solution considered a variable given by the difference between T0 and T30 in the number of (altered) dichotomous MS components and then categorized in: −1 if the subject had a worsening (at T30) in at least one factor, 0 if nothing changed at T30, and 1 if the subject experienced an improvement (at T30) in at least one factor. The latter solution considered the relative variation in each of the 5 MS factors (e.g., (triglycerides(T0)-triglycerides(T30))/triglycerides(T0), continuous variable). Such new variables were then categorized as follows: −1 if the subject experienced a worsening ≥5%, 1 if there was an improvement ≥5%, and 0 if there was no change or it was <5% in both directions. The 5% threshold was considered as the minimally relevant expected change given the study intervention. Given the small number of subjects generally having a worsening over time, in all the analyses, these were considered with those not experiencing any change or a very small (<5%) one. All the fitted models included the main effects for time and for the MS component change variable, as well as an interaction term between them. In this way, we could assess whether a different temporal trajectory in genus relative abundances was present between the subjects with and without any improvement in metabolic disorders. The ANOVA-type statistics (ATS) were considered for the interpretation of the results.

The ^1^H NMR spectra of urine (ZGCPPR Bucker standard pulse sequence) were data-reduced to equal length integral segments of 0.02 ppm bucket width considering the NMR spectral range 9.5–0.5 ppm for the bucketing process and multivariate analyses. Resonances of residual water (4.95–4.60 ppm) and urea (6.00–5.60 ppm) were discarded because of the variability (though limited) of urea signal and variations in the suppression of the water signal. Moreover, NMR signals of creatinine (4.08–4.03 and 3.07–3.03 ppm) and citrate (2.70–2.65 and 2.57–2.51 ppm) were combined to account for shifting signals [52] and the remaining buckets were then normalized to the total area to minimize differences in urine concentration between samples and subsequently mean-centered. For statistical analyses, all the imported data were mean-centered and divided by the square root of the standard deviation of each variable using the Pareto scaling algorithm. Unsupervised (blinded) investigation of the data was performed by Principal Component Analysis (PCA) and subsequently analyzed using Orthogonal Projections to Latent Structure Discriminant Analysis (OPLS-DA). In particular, using the NMR buckets as input variables, the PCA was preliminarily used to explore the potential differences in the metabolome profile at baseline and/or presence of outliers (95% confidence ellipse using Hotelling’s T^2^ statistics). OPLS-DA analysis was also performed on NMR bucket-reduced data, in which results were clearly discriminated in the first predictive t [1] component. The parameters calculated to assess the validity of the established models were the total amount of variation between and within the groups (R^2^Y and R^2^X) and the predictive ability of the models as determined by permutation test and seven-fold cross-validation (Q^2^). NMR discriminant variables were evaluated by the S-line Plots, identified with the loading scaled as a correlation coefficient value (p(corr)) of the OPLS-DA models.

Most of the calculations were performed using the open-source statistical computing environment R [53]. The dietary data were analyzed with libraries psych [54], cluster [55], cclust [56], and mclust [57]; the microbiome data were analyzed with libraries vegan (http://www.cran.r-project.org/package=vegan/, last accessed on 17 May 2021), Made4 [58] and nparLD [51]. Metabolome data were analyzed using SIMCA-14 software (Sartorius Stedim Biotech, Umeå, Sweden).

## 3. Results

### 3.1. Description of the Study Participants at Baseline

#### 3.1.1. Anthropometric, Biochemical and Immunological Characteristics

Participants were recruited between October 2018 and September 2019. Of the 67 subjects assessed for eligibility, 7 were excluded because did not meet the inclusion criteria. The analyses were therefore performed on 60 subjects, if not otherwise indicated.

Table 1 shows the baseline characteristics of the recruited subjects, altogether and separately for the two study groups. Most of the study subjects were females (78.3%) and the median age was 47 years [IQR: 12]. Nine subjects suffered from MS at baseline, (with 6 of them belonging to the SA-group and 3 of them belonging to the PROB-group). Percentages of participants in each adherence category (from the lowest to the highest one) to the IMI were equal to 40.7%, 39.0% and 20.3%, respectively. This is in line with previous literature on dietary patterns of Italian subjects from the EPICOR study, a prospective collaborative investigation of the causes of cardiovascular diseases in Italian volunteers recruited in 1993–1998 within the Italian section of EPIC (47,021 Italian men and women in total) [47]. No substantial differences were observed across SA- and PROB-groups in any examined variable, with the exception of age (higher in SA-group) and IL-17A (lower in SA-group), as compared to PROB-group (*p* = 0.015 and *p* = 0.004, respectively, Mann Whitney U test). However, the distribution of IL-17A was quite extreme: only 36% of the data were different from zero, with one subject showing a value higher than the 90th percentile of the overall distribution.

#### 3.1.2. Dietary Habits

The analysis of dietary data at baseline was based on 59 subjects, as 1 subject did not fill in most of the FFQ items, thus resulting in a total energy intake <500 kcal.

The distribution of study participants and of their baseline characteristics according to categories of adherence to Mediterranean diet, as measured by the IMI, are shown in Table 2. The distribution of baseline characteristics was similar across categories of adherence to the IMI (all *p*-values were nonsignificant).

Visual inspection, Bartlett’s test of sphericity (making it possible to reject the null hypothesis that the correlation matrix is the identity matrix with a *p* < 0.001), overall (0.84) and individual measures of sampling adequacy (20 nutrients with measures ≥0.90) suggested that the nutrient-based correlation matrix was adequate for EFA (Supplementary Table S1). Table 3 gives the factor-loading matrix for the three retained DPs.

The selected DPs explained ~80% of the total variance. Any nutrient had one or more factor loadings ≥0.30, thus suggesting that all the selected nutrients were relevant in this analysis. The greater the loading of a given nutrient to a factor was, the higher the contribution of that nutrient to the factor. The first DP was named “Animal products”, as it was characterized by high loadings on animal protein, cholesterol, niacin, zinc, saturated fatty acids, phosphorus, vitamin D, sodium, vitamin B6, retinol, riboflavin, thiamin, calcium, and linoleic acid. The second DP, named “Vitamins and fiber”, was characterized by high loadings on vitamin C, beta-carotene, total fiber, total folate, vitamin E, potassium, monounsaturated fatty acids, and soluble carbohydrates. The third DP, named “Regional”, had high loadings on vegetable protein, other polyunsaturated fatty acids, and starch. The communalities—measuring the proportion of each nutrient’s variance explained by the retained DPs altogether—were generally satisfactory, being greater or equal to 0.70, except for five nutrients (other polyunsaturated fatty acids, retinol, vitamin D, riboflavin and soluble carbohydrates). In addition, when considering Pearson correlation coefficients >0.45 with the amount of selected food groups on the same subjects, the “Animal products” DP score was positively correlated with (in order from the highest to the lowest coefficients) consumption of red meat (especially, beef and pork), offal, processed meat, fish, eggs, coffee, cheese, and olive oil; the “Vitamins and fiber” DP score had positive correlation coefficients with root vegetables, other (than citrus) fruit, olive oil, leafy vegetables (raw and cooked), cabbages, soups and bouillon, whereas the “Regional” DP was positively correlated with the consumption of grains (wholemeal), tea (including herbal tea), and leafy vegetables (raw and cooked).

Table 4 provides a description of the CLU-based DPs or clusters identified at baseline using the PAM CLU method on the EFA-based DP scores.

The optimal number of clusters was equal to four. Each cluster showed an extreme behavior (exceeding the third score quartile) in one of its center coordinates, except for cluster number 2 (C2) (19 subjects). Specifically, the C1 center was extreme on the “Regional” factor (11 subjects), and the C3 center was extreme on the “Animal products” factor (18 subjects), whereas the C4 center was extreme on the “Vitamins and fiber” pattern (11 subjects). The C2 coordinates were all lower than the corresponding factor medians, being close to the first quartile for the “Animal products” and “Vitamins and fiber” factors and being between the first quartile and the median of the “Regional” factor score: we therefore named C2 as “Low consumers”. Similarly, higher-than-median score coordinates described C1 for the remaining “Animal products” and “Vitamins and fiber” patterns; we indicated it as the “High consumers” cluster, especially extreme on the “Regional” DP. The extreme coordinate of the C3 center on the “Animal products” factor was balanced with approximately median score coordinates on the “Vitamins and fiber” and “Regional” factors, thus pointing to an “Omnivorous with meat prevalence” cluster. Finally, we named C4 as the “Omnivorous with plant-based foods prevalence” cluster: apart from the extreme coordinate on the “Vitamins and fiber” pattern, the remaining coordinates were both close to—or even lower than—the corresponding first quartile of the factor score.

The identified clusters were similar with respect to demographic, anthropometric, biochemical, and immunological characteristics (data not shown).

#### 3.1.3. Gut Microbiota Profiling

The gut microbiota of the enrolled subjects was profiled at baseline and during the intervention at five time points (see Figure 1), for a total of 343 fecal samples subjected to 16S rRNA gene sequencing. Seventeen samples were missing or of low quality. A total of 6,682,079 high-quality reads (mean ± SD, 19,481 ± 9578) were obtained and analyzed.

**Figure 2:**
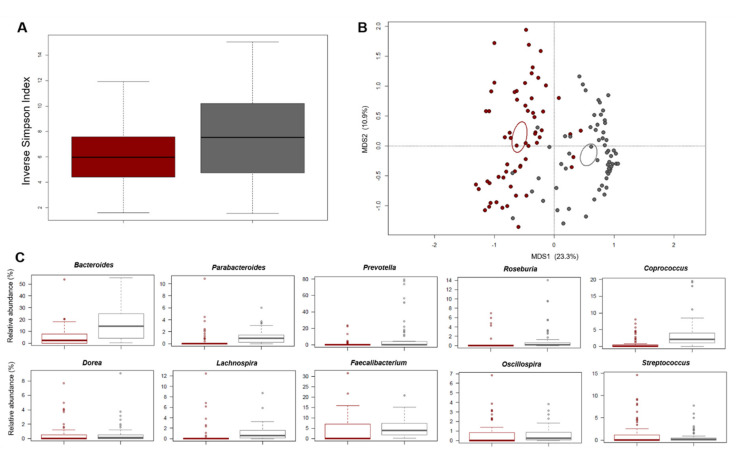
The gut microbiota of study subjects at risk for metabolic syndrome segregated from those of healthy Italian controls, matched by microbiota-associated confounding factors (i.e., age and gender). (**A**) Boxplots showing the distribution of alpha diversity, according to the inverse Simpson index, in study subjects (dark red) compared to healthy Italian controls (grey). A significantly reduced diversity was observed in the former group (*p* = 0.01, Wilcoxon test). (**B**) PCoA plot of beta diversity, based on Bray–Curtis dissimilarity between the genus-level microbial profiles. A significant separation between study subjects and healthy Italian controls was found (*p* = 0.001, permutation test with pseudo-F ratio). Samples are identified with colored dots as in (**A**). Ellipses include 95% confidence area based on the standard error of the weighted average of sample coordinates (dark red, subjects at risk for metabolic syndrome; grey, healthy controls). (**C**) Boxplots showing the relative abundance distribution of differentially represented genera between study subjects and healthy Italian controls (*p* ≤ 0.05, Wilcoxon test).

The baseline profile was compared with that of 60 healthy Italians from previous studies, matched by age and gender [42,43,44], which are well-known microbiota-associated confounding factors [59]. According to the inverse Simpson index, alpha diversity was significantly lower in the enrolled subjects than in the healthy controls (*p* = 0.01, Wilcoxon test) (Figure 2A).

Similarly, the PCoA of beta diversity, based on Bray–Curtis dissimilarity between the genus-level profiles, showed significant separation between the study samples and the healthy controls (*p* = 0.001, permutation test with pseudo-F ratio) (Figure 2B). In line with the available literature on gut microbiota in metabolic disorders [15,60,61,62], the study subjects showed a higher relative abundance of *Coriobacteriaceae* (*p* < 0.001, Wilcoxon test) and *Streptococcus* (*p* = 0.01), as well as reduced proportions of *Bacteroidaceae* members, including *Parabacteroides* (*p* < 0.001) (Figure 2C and Supplementary Figure S1). Interestingly, *Parabacteroides* has recently been suggested as a novel probiotic taxon for reducing obesity, inflammation levels and insulin resistance [63]. As expected [64], several health-associated SCFA-producing commensals belonging to the *Lachnospiraceae* and *Ruminococcaceae* families, including *Roseburia*, *Coprococcus*, *Lachnospira*, *Oscillospira* and *Faecalibacterium*, were also underrepresented in the gut microbiota of the study participants (*p* < 0.001).

Correlations between the relative abundances of bacterial taxa and anthropometric, biochemical and immunological parameters in the study subjects were next sought (Supplementary Figure S2). Despite the low correlation coefficients, it is worth noting that a *Coriobacteriaceae* member (i.e., *Adlercreutzia*) correlated positively with total cholesterol (tau = 0.239, *p* = 0.03, Kendall rank correlation test) and LDL cholesterol (tau = 0.237, *p* = 0.03), while a negative correlation was found between *Akkermansia*, a mucus degrader associated with improved metabolic health [65] and insulin (tau = −0.226, *p* = 0.03). Furthermore, we found inverse correlations for *Bifidobacterium* (tau = −0.221, *p* = 0.04) and *Bacteroides* (tau = −0.216, *p* = 0.03) against IL-17A, as well as for *Bacteroides* (tau = −0.25, *p* = 0.02) and *Ruminococcus* (tau = −0.219, *p* = 0.02) against IFN-γ. *Ruminococcus* was also inversely correlated with IL-6 (tau = −0.215, *p* = 0.02).

As for associations with dietary habits (Supplementary Figure S3), the Bray–Curtis-based PCoA showed a significant separation between the microbiota structure of the “Omnivorous with plant-based foods prevalence” cluster and the others (*p* = 0.05, permutation test with pseudo-F ratio). When looking for a potential relationship with food groups, we found that consumption of milk (*p* ≤ 0.05, “envfit” function) and white meat (*p* ≤ 0.1) was associated, or tended to be, with the microbiota of individuals from the “High consumers”, “Low consumers”, and “Omnivorous with meat prevalence” clusters, where most animal products were represented to a greater or lesser extent. On the other hand, the microbiota of the “Omnivorous with plant-based foods prevalence” cluster subjects tended to be associated with the consumption of garlic and onion, and butter (*p* ≤ 0.1). At the taxonomic level, the “High consumers”-related gut microbiota was characterized by greater proportions of *Enterobacteriaceae* members (*p* = 0.02, Kruskal-Wallis test) and a tendency to higher amounts of *Bifidobacterium* (*p* = 0.1), a well-known probiotic taxon associated with dairy consumption. The “Omnivorous with plant-based foods prevalence”-related microbiota tended to be discriminated by greater relative abundances of *Blautia* and *Butyricimonas* (*p* = 0.1). It is worth mentioning that both genera are SCFA producers, even if the former is acetogenic and the latter butyrogenic. However, conflicting data exist on the association between *Blautia* and metabolic health, with particular reference to abdominal fat [66,67], and its abundance was found to be positively associated with saturated and monounsaturated fatty acids [13], probably suggesting the existence of different oligotypes with various metabolic capacities.

#### 3.1.4. Urine Nuclear Magnetic Resonance-Based Metabolomics

The urine metabolome was profiled at baseline and after intervention (see Figure 1), for a total of 120 urine samples. Six NMR spectra were excluded due to the presence of detectable ethanol as contaminant and high levels of glucose, thus obtaining a total of 114 urine samples suitable for successive multivariate analyses. Although very complex, the ^1^H NMR spectra of urine contained thousands of sharp lines from predominantly low-molecular weight metabolites. Resonances were directly assigned on their chemical shifts, signal multiplicities (resolved by 2D NMR experiments, randomly performed on urine samples) and literature data [45]. Based on all subjects together, the main metabolites identified were creatinine, trimethylamine-N-oxide (TMAO), glycine, citrate, alanine, acetate, erythritol, trigonelline and hippurate. No substantial differences in the metabolomic profile between the two study groups and among the dietary clusters were observed. Moreover, samples were also homogeneous with respect to the information reported in Table 1 (Supplementary Figure S4).

As for associations with the gut microbiota (Supplementary Figure S2), again the correlation coefficients were very small, but it is interesting to mention that we found a negative correlation between the relative abundance of *Lactobacillus* and several metabolites, i.e., creatinine (tau = −0.234, *p* = 0.04), TMAO (tau = −0.226, *p* = 0.05) and phenylacetylglycine (tau = −0.239, *p* = 0.03). A positive correlation was found between *Blautia* and trigonelline (tau = 0.219, *p* = 0.03), an alkaloid with potential anti-diabetic activity [68].

### 3.2. Effects of the Dietary Intervention

#### 3.2.1. Impact on Anthropometric, Biochemical and Immunological Parameters

Supplementary Table S2 shows the comparison of anthropometric, biochemical and immunological characteristics, as well as presence of MS, for all enrolled subjects and by SA- and PROB-groups, at the two time points T0 and T30. Compared to the baseline, some parameters changed after the dietary intervention. In particular, a statistically significant reduction in insulin values was observed (*p* = 0.013, Wilcoxon signed rank test). Other measures, such as cortisol, blood pressure (BP), and body mass index (BMI), showed an improvement, that is, a decrease, after the intervention, even if not statistically significant at a 5% level. Similarly, MS was detected in six of the nine subjects with the disease at baseline, thus representing a statistically significant improvement from the baseline to the end of the intervention (*p* < 0.001, McNemar test). When inspecting group-specific differences, statistically significant reductions were observed for systolic BP in the SA-group (*p* = 0.032, paired *t*-test), and for cortisol and insulin in PROB-group (*p* = 0.020 and *p* = 0.006, Wilcoxon signed rank test, respectively) (Supplementary Figure S5). With respect to insulin, one subject reported a very high value at baseline. However, after removal of this individual, the difference remained statistically significant (*p* = 0.010). In the SA-group, a slightly lower BMI was registered after the intervention (medians equal to 26.1 [IQR: 5.1] and 25.4 [IQR: 5.9] kg/m^2^ for T0 and T30, respectively; *p* = 0.057, paired *t*-test), as well as reduced glucose levels (medians equal to 83.0 [IQR: 14.0] and 83.0 [IQR: 8.0] mg/dL for T0 and T30, respectively; *p* = 0.067, Wilcoxon signed rank test). In addition, in both groups, the number of subjects with MS decreased from the baseline to the end of the intervention, in a statistically significant way (SA-group: from six to four, *p* < 0.001, McNemar test; PROB-group: from three to two, *p* = 0.002, McNemar test). No other significant modifications were observed (data not shown).

#### 3.2.2. Impact on the Gut Microbiota Composition

No differences in alpha and beta diversity were observed over time in the SA-group (Supplementary Figure S6). Similar results (i.e., no separation between fecal samples at different time points in the PCoA of beta diversity) were obtained in the PROB-group, for which, however, a temporal reduction in alpha diversity was found, with the lowest values after 30 days of intervention (Faith’s Phylogenetic Diversity and number of observed ASVs: *p* ≤ 0.04, Friedman test) (Supplementary Figure S6).

**Figure 3:**
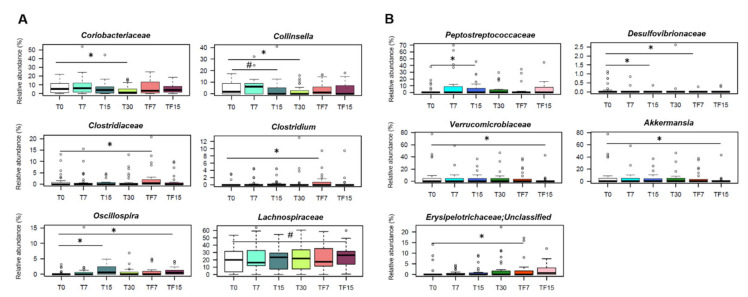
Impact on the gut microbiota of a diet with fresh foods from organic symbiotic agriculture versus probiotic supplementation. Boxplots showing the relative abundance distribution of differentially represented taxa over time, in subjects at risk for metabolic syndrome consuming fresh foods from organic symbiotic agriculture (SA-group) (**A**), or receiving probiotic supplementation (PROB-group) (B). The gut microbiota was profiled at baseline (T0), after 7 (T7), 15 (T15) and 30 (T30) days of intervention, and at follow-up, 7 (TF7) and 15 (TF15) days after the end of the intervention. *, *p* ≤ 0.05; ^#^, 0.05 < *p* ≤ 0.1; Wilcoxon test.

Interestingly, at the compositional level, some of the dysbiotic features identified at baseline were reversed after intervention with SA-derived foods, and others tended to be reversed (Figure 3A).

In particular, the relative abundance of *Coriobacteriaceae*, especially *Collinsella*, a potential pathobiont proposed as a target in future microbiome-based interventions for metabolic disorders [69], was significantly reduced in SA-group after 30 days of diet (*p* ≤ 0.007, Wilcoxon test), with proportions of *Collinsella* tending to decrease already after 15 days (*p* = 0.1). Furthermore, we observed a rapid increase in the relative abundance of *Oscillospira* (T0 vs. T15, *p* = 0.02), a likely heritable taxon positively associated with leanness and health [70]. It is worth noting that such an increase persisted in the follow-up (T0 vs. TF15, *p* = 0.04), while other changes appeared only later on, namely the increase in *Clostridiaceae* (T0 vs. TF7, *p* = 0.02) and the tendency towards increased amounts of *Lachnospiraceae* (T0 vs. TF15, *p* = 0.07). For these taxa, the baseline variation in the 2 weeks prior to dietary intervention was not significant (*p* > 0.05, Friedman test) (Supplementary Figure S7), which supports that the aforementioned compositional changes were related to the change in diet and not to the typical oscillations of the gut microbiota in the absence of perturbation (see [71] for a recent discussion on the topic). When looking at the compositional variations within the four dietary clusters (Supplementary Figure S8), we found that “High consumers” cluster individuals showed a significant decrease in *Desulfovibrio* after 7 days of diet (*p* = 0.04, Wilcoxon test), which persisted over time, and a tendency towards increased proportions of *Roseburia* at the end of the intervention, until follow-up (*p* = 0.1, Friedman test). On the other hand, for the “Low consumers” and the “Omnivorous with meat prevalence” clusters, we found increasing trends in other SCFA producers, i.e., *Coprococcus* (*p* = 0.06) and *Oscillospira* (*p* = 0.09), respectively. Interestingly, *Coprococcus* correlated negatively with cortisol, whose levels decreased after intervention as reported above (Supplementary Figure S9). A negative correlation in the whole dataset was also found between *Phascolarctobacterium*, another SCFA (mainly propionate) producer, and blood pressure, in line with previous studies associating it with improved metabolic health [72]. Furthermore, the relative abundance of some taxa and precisely *Adlercreutzia*, *Prevotella*, *Butyricimonas* and *Blautia*, showed overall expected correlations with total cholesterol, LDL cholesterol and waist-to-hip ratio in all sample (Supplementary Figure S9).

As for the PROB-group, apart from the reduction in the proportions of *Desulfovibrionaceae* (T0 vs. T15 and T0 vs. TF7, *p* = 0.04, Wilcoxon test), we observed some unfavorable changes, including the increase in pro-inflammatory taxa, such as *Peptostreptococcaceae* (T0 vs. T15, *p* = 0.02) and unclassified *Erysipelotrichaceae* members (T0 vs. TF7, *p* = 0.02), and the decrease in the metabolic health-associated genus, *Akkermansia* (T0 vs. TF15, *p* = 0.04) (Figure 3B). Again, these taxa showed no significant changes in the 2 weeks prior to intervention (*p* > 0.05, Friedman test) (data not shown).

When we inspected the relationship between changes in genus relative abundances and improvements in MS components over time within the SA-group, we identified two genera that showed peculiar trends: *Oscillospira* and *Akkermansia* (Supplementary Figure S10). The former showed an increasing trend over time (*p* = 0.01, ATS test for time effect), although similar in subjects who had an improvement in at least one MS factor and in those who didn’t have it (*p* = 0.14, ATS test for group effect). The latter showed a difference between the two mentioned groups at T0, with lower values for those showing an improvement in the MS factors. However, this difference tended to disappear later on due to a subsequent increase of *Akkermansia* relative abundance over time for this group (*p* = 0.08, ATS test for the interaction effect). In line with the available literature [65], this further stresses the close association of *Akkermansia* with metabolic health.

The same approach was then applied to the single components of MS. Within the SA-group, *Oscillospira* showed a consistent increasing temporal trend for each MS component (all *p* ≤ 0.01, ATS test for the time effect). An increase over time was also observed for *Roseburia* in relation to systolic BP (*p* = 0.07, ATS test for the time effect); however, this was similar for those who showed or not an improvement of at least 5% in this parameter (*p* = 0.65, ATS test for the interaction effect). Finally, *Lachnospira* showed a differential trend over time in relation to triglycerides and glycaemia, with an increase in those who improved only at the last time-point, TF15 (*p* = 0.04, ATS test for the interaction effect). When we analyzed PROB-group, other interesting trends were found. Among them, *Akkermansia* behaved differently between subjects who then showed or not an improvement in triglyceride levels, with an increase in the former up to T30 (*p* = 0.001, ATS test for the interaction effect). Differential trends were also observed for *Oscillospira* in relation to both triglycerides and glycaemia, with an increase in follow-up for those who showed reduced triglyceride levels (*p* = 0.01, ATS test for the interaction effect) and at T15 for those with decreased blood glucose (*p* = 0.03, ATS test for the interaction effect) (data not shown).

#### 3.2.3. Impact on the Urine Metabolome

Within the SA-group, after the dietary intervention, a good separation of the data points was observed as well as time-dependent discriminant metabolites. In particular, the corresponding S-line plot from OPLS-DA analysis showed increased levels of erythritol (3.78–3.68 ppm), glycine (3.57 ppm), citrate (2.68–2.54 ppm), acetate (1.93 ppm), and alanine (1.48 ppm), in samples after 30 days of treatment, with a concomitant reduction in the level of creatinine (4.06 ppm) and TMAO (3.27 ppm) (Supplementary Figure S11). Moreover, by comparing the predictive performances of the OPLS-DA models built for each dietary cluster, we observed that the “Omnivorous with meat prevalence” cluster showed a greater sample classification capacity (Q^2^ of 0.18) as compared to the others. This indicates that the intervention with SA-foods had a greater overall impact on subjects with “Omnivorous with meat prevalence” behavior. Interestingly, a relatively higher level of hippurate and a particularly pronounced decrease in TMAO level were observed in these subjects after intervention. The decrease of TMAO is extremely favorable, as this molecule, which is formed in the liver from trimethylamine, a metabolite synthesized by the gut microbiota from dietary choline, is recognized as a cardiovascular risk factor and associated with various negative health outcomes [73]. Regarding glycine, its circulating levels have been reported to decrease in metabolic disorders associated with obesity [74]. Moreover, plasma glycine concentration is altered according to food choice, being higher in vegetarian and vegan groups than in meat eaters [75].

Interestingly, some of the metabolites whose levels varied following the intervention showed consistent correlations with the proportions of some of the aforementioned microorganisms (Supplementary Figure S9). In particular, in the SA-group, erythritol correlated negatively with *Collinsella* (tau = −0.204, *p* = 0.04, Kendall rank correlation test) while positively with *Coprococcus* (tau = 0.25, *p* = 0.01). Positive correlations were also observed for *Coprococcus* (tau = 0.249, *p* = 0.01) and *Faecalibacterium* (tau = 0.246, *p* = 0.01) against alanine, as well as between *Faecalibacterium* and glycine (tau = 0.263, *p* = 0.009). Finally, as expected based on its metabolic propensity, *Blautia* positively correlated with acetate (tau = 0.187, *p* = 0.05).

Within the PROB-group, a good sample discrimination after treatment was observed. In particular, the corresponding S-line plot of the OPLS-DA analysis (Supplementary Figure S11) allowed us to identify a decreased level of trigonelline (8.81–8.06 ppm, detectable only in the “High consumers” cluster). The analyses within dietary clusters did not reveal significant deviations, with the exception of relatively higher levels of hippurate in the “High consumers” and “Omnivorous with meat prevalence” clusters (data not shown).

## 4. Discussion

In this first pilot intervention study offering SA-derived products to subjects at risk for MS, we showed that mycorrhized farming products modulate certain components of the gut microbiota; this effect was accompanied by changes in some metabolic parameters and urinary metabolites and it was partly modulated by DPs at baseline. In addition, two out of six study participants suffering from MS at baseline no longer had MS after the intervention.

In line with the existing literature on gut microbiota in metabolic disorders [15,61,62], the study subjects, as compared to healthy age/sex-matched Italian adults, showed some dysbiotic features at baseline, namely: (i) reduced biodiversity; (ii) lower proportions of health-associated taxa, mainly SCFA producers from the *Lachnospiraceae* and *Ruminococcaceae* families, i.e., *Lachnospira*, *Coprococcus*, *Roseburia*, *Oscillospira* and *Faecalibacterium*, as well as *Parabacteroides*; and (iii) greater relative abundance of generally subdominant taxa with pathogenic potential, such as *Coriobacteriaceae* and *Streptococcus*. While the decrease in SCFA producers is frequently found in disparate diseases and pre-disease conditions, as a probably universal dysbiotic signature [64], the overrepresentation of *Coriobacteriaceae* could be regarded as a potentially specific alarm bell for metabolic disorders. Indeed, increased levels of *Coriobacteriaceae* members have been found in conditions of overweight and obesity, as well as in the context of type 2 diabetes and symptomatic atherosclerosis, and directly associated with metabolic risk factors, including those predisposing to MS, such as insulin, triglycerides and LDL cholesterol [15,17,60,76], as also observed in our sample. It has been hypothesized that a gut microbiota profile enriched in such microbes may influence intestinal absorption of cholesterol, hepatic glycogenesis and triglyceride synthesis, as well as interfere with the expression of tight junction proteins, resulting in loss of barrier integrity, metabolic endotoxemia and chronic low-grade inflammation [17,77]. On the other hand, *Parabacteroides*, which was found to be underrepresented in study participants, has recently been proposed as a probiotic candidate for its metabolic benefits, as observed in mouse models, probably through the production of succinate and secondary bile acids [63,78].

Dietary habits at baseline were well characterized by the use of DPs, which are combinations of dietary components meant to summarize total diet—or key aspects of the overall diet—in free-living individuals, as measured at one or more time points. As compared to analyzing single dietary components one at a time, the DP approach allows to capture well-known interactive effects among nutrients or foods, while solving statistical issues related to collinearity between food components and adjustment for multiple comparisons [79,80]. In the current application, we referred to both a priori (or index-based) and a posteriori (empirically derived) DPs [80]. Among available a priori DPs, we referred to the IMI to assess if study participants did follow a Mediterranean diet and to what extent it happened. We showed that our study sample adhered to the Mediterranean diet—as measured by the IMI—to the same extent as the more representative Italian sample of subjects belonging to the EPICOR study [47]. In addition, we applied a combination of EFA and CLU for identifying a posteriori DPs at baseline and relating them to microbiome or metabolome. The small number of subjects—as compared to nutrients—and the by-product of having the correlation structure of nutrients described in terms of EFA-based DPs suggested to perform an EFA before CLU. Cluster-based DPs provided an additional advantage within this project. As individual dietary habits were summarized with one belonging indicator—and not by multiple factors simultaneously—the assessment of the potential links between diet and microbiome or diet and metabolome was easier with CLU-based DPs rather than with the more common EFA based ones. In addition, the current paper explores the use of a more robust CLU partitioning algorithm, PAM, which is more suitable than the well-known k-means. The DPs derived from the application of EFA and CLU are very similar to those derived in an Italian network of case-control studies exploring the association between diet and cancer at several sites (e.g., [48,49,81]); the statistical approach was similar and the same Italian Food Composition Tables [34] were used to convert food items into nutrients, thus improving the possibility of finding similar patterns, as far as they are indeed present. In detail, our EFA-based “Animal products” DP—loading high on animal protein, fats, zinc, B-group vitamins, calcium, phosphorus, sodium, vitamin D, and retinol—was mostly overlapping with the corresponding “Animal products” identified in the previous network (e.g., [48,49,81]); minor differences between the two DPs likely included the major role of meat, including offal—represented by the additional presence of retinol—in our DP, as compared to the major role of dairy products—represented by the highest loadings on calcium and phosphorus—identified on similar DPs in the case-control studies. Similarly, our EFA-based “Vitamins and fiber” DP—loading high on vitamin C, beta-carotene, total fiber, total folate, vitamin E, potassium, monounsaturated fatty acids, and soluble carbohydrates—was similar to the corresponding “Vitamins and fiber” DP identified in the network, with both DPs pointing to consumption of fruit and vegetables; a minor difference between the 2 DPs dealt with the less dominant role of the “citrus fruits” food group in the current study, as compared to the case-control study network. Moreover, in our “Vitamins and fiber” DP, we have oils and vegetable consumption together (with high loadings on vitamin E and monounsaturated fatty acids), in the absence of an additional DP targeting vegetable fats. The “Regional” DP identified in the current study—loading high on vegetable protein, other polyunsaturated fatty acids, and starch—is in between the “Starch-rich” and the “Animal unsaturated fatty acids” DPs, as it combines vegetable protein and starch (but not sodium) from the formed DP with the other polyunsaturated fatty acids (but not vitamin D and niacin) from the latter DP. A paper of the same network applied CLU on the factor scores from a previous EFA [82]; like in the current application, each of the five selected clusters showed an extreme behavior in one of the center coordinates, except for one that is similar to our “Low consumers” DP. Specifically, two cluster centers were extreme on a “Vitamins and fiber” and on an “Animal products” DP that were comparable to our “Omnivorous with plant-based foods prevalence” and “Omnivorous with meat prevalence”, respectively; the remaining two cluster centers were extremes on an “Unsaturated fats” and on a “Starch-rich” DP, which combine elements of our “High consumers” DP, although the fat profile is likely more oriented towards the vegetable source in the previous paper [82]. Within the Italian arm of the EPIC Elderly project, four EFA-based DPs (21% of explained variance) were identified on a comparable population interviewed with the same FFQ used in the current study [83,84]. Among them, the “prudent” (cooked vegetables, pulses, cabbage, seed oil and fish) and the “olive oil & salad” (raw vegetables, olive oil, soup and chicken) share similarities with our “Vitamins and fiber” DP, although we did not observe the simultaneous presence of vegetables and meat; their “pasta & meat” (pasta, tomato sauce, red meat, processed meat, bread and wine) is in between our “Animal products” and “Regional” DPs, but we were not able to identify any sort of “sweet & dairy” (sugar, cakes, ice cream, coffee and dairy) DP in our study sample. In conclusion, our study provided the possibility to confirm that, to some extent, Italian DPs derived with multivariate statistics show a good reproducibility across studies [85].

When looking for associations between CLU-based DPs and the gut microbiota, we found that the basal microbiota structure of the “Omnivorous with plant-based foods prevalence” cluster separated from all others, being discriminated by higher proportions of *Blautia* and *Butyricimonas*. Both genera are producers of SCFAs (mainly acetate and butyrate, respectively), which could play a multifactorial role in maintaining metabolic and immunological homeostasis [86]. With specific regard to *Blautia*, although the data have not been replicated in the elderly population [67], this taxon has recently been found to be inversely associated with visceral fat in a large population-based adult cohort [66], and hypothesized to have the potential to counteract MS risk factors. Unlike that study, in which no dietary factor correlated with *Blautia* amount, here we found that the *Blautia* and *Butyricimonas*-enriched “Omnivorous with plant-based foods prevalence” cluster was particularly associated with garlic, onion and butter consumption, suggesting a possible link between these foods and those microorganisms. However, as far as we know, no information is currently available on their impact on the microbiota, except for a correlation between *Blautia* and saturated and monounsaturated fatty acids [13], which are the major lipids of butter. As for the other clusters, it is worth mentioning that the “High consumers”-related microbiota was discriminated by higher proportions of enterobacteria and bifidobacteria, and associated with the intake of animal products, such as meat and milk. This was expected, as the link between *Bifidobacterium* and the intake of dairy products is well established, from early childhood [87,88]. After one month of dietary intervention, study participants experienced modest improvements in BMI, insulin and cortisol levels (especially in PROB-group), and BP (particularly in SA-group).

As for gut microbiota, some dysbiotic signatures were reversed and others tended to be reversed after intervention. In particular, the SA-based diet counteracted the increase in pathobionts, namely *Coriobacteriaceae*, as well as the decrease in SCFA producers, i.e., *Lachnospiraceae* and *Oscillospira*, a potentially heritable taxon capable of promoting leanness [70]. Interestingly, the increase in *Oscillospira* was already noticeable after only 15 days of diet, as well as a tendency towards reduced proportions of *Collinsella*, the dominant taxon of the *Coriobactaeriaceae* family, thus suggesting a modulatory effect even in the short term. Since these taxa did not show significant changes over the run-in period, we can reasonably argue that their variation is the result of introducing SA-derived food products into the diet. As expected, the extent of microbiota modulation was greater in participants not belonging to the “Omnivorous with plant-based foods prevalence” cluster, for whom the increase in *Lachnospiraceae* and *Ruminococcaceae* members, along with the decrease in *Desulfovibrio* (a sulphate-reducing pathobiont found to be increased in type 2 diabetic patients and those suffering from inflammatory bowel disease [89,90]) were more evident. Furthermore, it should be noted that an increase in *Akkermansia* specifically discriminated the participants who showed improvement in at least 1 risk factor for MS, further stressing the close association between this taxon and metabolic health [17,65]. *Akkermansia* is in fact a mucus degrader with promising metabolic benefits, as validated in a recent proof-of-concept exploratory study [65]. On the other hand, in the PROB-group, i.e., in subjects receiving probiotic supplementation, we observed a reduction of sulphate-reducing bacteria, but also several unfavorable microbiota changes, including reduced diversity and relative abundance of *Akkermansia.* In this group, we also found increased proportions of *Peptostreptococcaceae* and *Erysipelotrichaceae*, less characterized microorganisms, but generally associated with increased inflammatory tone [91,92].

Urine metabolomics confirmed a general beneficial effect of SA-derived products on the metabolic health of the participants, as exemplified by the decrease in TMAO levels and the increase in citrate levels in SA-group. As a result of the microbiota–host co-metabolism of dietary choline, TMAO has indeed been repeatedly associated with cardiovascular disease risk and atherosclerosis [73]. As for citrate, its urinary excretion rate mainly depends on the acid–base status of the body, and urinary citrate has long been recognized as an inhibitor of calcium salt crystallization [93]. Even small acid loads, such as meat-based or protein-rich meals in general, reduce urinary citrate excretion. It is, therefore, tempting to speculate that the intervention in SA-group may have a more protective role against kidney disease. Furthermore, subjects from the “Omnivorous with meat prevalence” cluster in both SA-group and PROB-group, and those from the “High consumers” cluster in PROB-group showed a relatively higher level of hippurate following the intervention. Hippurate has been strongly associated with increased gut microbiome diversity, consumption of polyphenol-rich foods, and reduced odds of MS [94]. On the other hand, subjects from the “High consumers” cluster of PROB-group experienced a lower amount of urine trigonelline, which could be unfavorable. Despite a possible dietary-related origin, trigonelline is mostly biosynthesized by the gut microbiota during the conversion of S-adenosylmethionine to S-adenosylhomocysteine (methionine cycle). Interestingly, this metabolite has been inversely correlated with obese and diabetic phenotypes [95].

This study has several strengths, including: (i) the collection of data over time, allowing for a strict control of potential changes over the study period; (ii) the collection of anthropometric, biochemical, and immunological information, as well as baseline dietary data, which allowed for a parallel exploration and interpretation of temporal effects in microbiome and metabolome; and (iii) the use of a validated instrument for the assessment of subjects’ DPs. However, the present study has also some limitations: (i) its small sample size, even if legitimate for a hypothesis-generating study characterized by considerable organizational commitment; (ii) the lack of a control group who eat the same products of the SA-group but produced with conventional techniques, to disentangle the actual contribution of SA-based products; and (iii) the lack of an assessment of individual adherence to the dietary intervention to monitoring also the evolution of diet quality and potential shifting towards more balanced or controlled diets, including a higher adherence to the Mediterranean diet, which is already known to provide some beneficial effects in obesity, type 2 diabetes, cardiometabolic disease risk and aging [15,96,97,98].

## 5. Conclusions

To our knowledge, this is the first study exploring the potential beneficial effects of SA-derived products on the gut microbiota and urinary metabolome in humans. Our preliminary evidence points to some improvements in the amounts of certain microorganisms and metabolites relevant to health, as well as in some risk factors for MS, in subjects receiving fresh food products. These benefits were greater in those who followed less healthy dietary habits. Participants receiving probiotics also showed some changes in microbial, metabolic and health parameters but the effects were marginal and not entirely favorable, in accordance with recent literature [99]. Diets based on foods from organic symbiotic crops may therefore be effective in modulating unbalanced microbiomes towards eubiotic configurations, and improving metabolomics profiles and metabolic health, with likely lower risk of developing MS and related disorders. Future studies in larger cohorts or randomized controlled trials, possibly including patients with MS or other metabolic diseases, and employing other omics techniques, such as shotgun metagenomics, are needed to validate these findings and provide further functional insights into the SA-based diet–microbiota–host axis.

## Figures and Tables

**Table 1:** Distribution at baseline of anthropometric, biochemical, and immunological characteristics for all study participants and separately for the dietary intervention groups. Italy, 2018–2019.

	All (*n =* 60)		SA-Group (*n =* 33)		PROB-Group (*n =* 27)		*p*
	Median	[Min–Max]	Median	[Min–Max]	Median	[Min–Max]	
Gender, *n* (%)							0.176
Male	13	(21.7)	5	(15.2)	8	(29.6)	
Female	47	(78.3)	28	(84.8)	19	(70.4)	
Smoking habit, *n* (%) ^1^							0.860
Never smoker	26	(48.1)	14	(46.7)	12	(50.0)	
Ex-smoker	22	(40.7)	12	(40.0)	10	(41.7)	
Current smoker	6	(11.1)	4	(13.3)	2	(8.3)	
Age at enrollment, years	46.9	[18.3–86.4]	52.7	[34.6–86.4]	45.3	[18.3–64.2]	0.015
Weight, kg	70.5	[44.0–103.0]	70.0	[44.0–103.0]	72.0	[47–94.5]	0.953
Height, m	1.65	[1.4–1.8]	1.7	[1.4–1.8]	1.7	[1.5–1.8]	0.183
BMI, kg/m^2^	25.7	[19.2–36.8]	26.1	[19.2–36.8]	25.3	[19.8–33.3]	0.427
Waist circumference, cm	85.0	[64.0–113.0]	85.0	[67.0–113.0]	84.0	[64.0–102.0]	0.639
Hip circumference, cm	105.0	[89.0–123.0]	105.0	[89.0–123]	104.0	[90.0–116.0]	0.312
WHR	0.8	[0.7–1.0]	0.8	[0.7–1.0]	0.8	[0.7–1.0]	0.783
Abdomen circumference, cm	98.5	[69.0–120]	98.0	[78.0–120.0]	99.0	[69.0–111.0]	0.582
Glucose, mg/dL	82.5	[66.0–212.0]	83.0	[66.0–212.0]	82.0	[72.0–103.0]	0.271
Cholesterol, mg/dL ^1^	193.0	[136.0–269.0]	190.0	[136.0–269.0]	195.0	[139.0–269.0]	0.345
HDL, mg/dL ^1^	59.0	[31.0–94.0]	65.0	[31.0–94.0]	55.0	[34.0–86.0]	0.061
LDL, mg/dL ^1^	113.0	[55.0–171.0]	106.0	[67.0–171.0]	119.0	[55.0–167.0]	0.064
Triglycerides, mg/dL ^1^	90.0	[43.0–365.0]	91.5	[43.0–365.0]	90.0	[44.0–243.0]	0.879
Cortisol, µg/L ^1^	125.0	[61.0–268.0]	124.5	[68.0–206.0]	129.0	[61.0–268.0]	0.744
Insulin, mU/L ^1^	9.2	[3.0–93.3]	8.9	[3.0–28.2]	10.1	[5.1–93.3]	0.169
Systolic BP, mmHg ^1^	120.0	[97.0–155.0]	120.0	[100.0–155.0]	115.0	[97.0–150.0]	0.072
Diastolic BP, mmHg ^1^	70.0	[55.0–90.0]	70.0	[60.0–90.0]	70.0	[55.0–90.0]	1.000
MS, *n* (%) ^1^							0.488
No	50	(84.7)	26	(81.2)	24	(88.9)	
Yes	9	(15.3)	6	(18.8)	3	(11.1)	
INF-γ ^1^	0	[0.0–7.5]	0	[0–2.8]	0	[0–7.5]	0.646
IL-6 ^1^	1.3	[0.0–254.4]	1.3	[0–55.5]	1.6	[0–254.4]	0.613
IL-10 ^1^	0.3	[0.0–15.0]	0	[0–15.0]	0.6	[0–4.5]	0.087
IL-17A ^1^	0	[0.0–18.8]	0	[0–2.6]	0.8	[0–18.8]	0.004
TNFα ^1^	0.2	[0.0–67.9]	0	[0–67.9]	0.3	[0–11.9]	0.419
IMI categories, *n* (%) ^1^							0.265
0–3	24	(40.7)	16	(50.0)	8	(29.6)	
4–5	23	(39.0)	10	(31.3)	13	(48.2)	
6–8	12	(20.3)	6	(18.7)	6	(22.2)	

BMI: body mass index; WHR: waist-to-hip ratio; HDL: high-density lipoprotein; LDL: low-density lipoprotein; BP: blood pressure; MS: metabolic syndrome; IMI: Italian Mediterranean Index. ^1^ With the exception of smoking habit, missing values were present only for one patient.

**Table 2:** Distribution at baseline of anthropometric, biochemical, and immunological characteristics by Italian Mediterranean Index tertiles (*n* = 59). Italy, 2018–2019.

	Low Adherence (Index Range: 0–3) (*n* = 24, 40.7%)		Medium Adherence (Index Range: 4–5) (*n* = 23, 39.0%)		High Adherence (Index Range 6–8) ^2^ (*n* = 12, 20.3%)		*p*
	Median	[Min–Max]	Median	[Min–Max]	Median	[Min–Max]	
Gender, *n* (%)							0.848
Male	5	(20.8)	6	(26.1)	2	(16.7)	
Female	19	(79.0)	17	(73.9)	10	(83.3)	
Smoking habit, *n* (%) ^1^							0.797
Never smoker	11	(50.0)	10	(47.6)	5	(45.5)	
Ex-smoker	8	(36.4)	10	(47.6)	4	(36.4)	
Current smoker	3	(13.6)	1	(4.8)	2	(18.2)	
Age at enrollment, years	46.2	[18.3–86.4]	53.7	[35.4–84.8]	46.1	[40.5–55.0]	0.389
Weight, kg	73.5	[47.0–103.0]	67.0	[44.0–94.5]	66.5	[56.0–84.5]	0.431
Height, m	1.7	[1.43–1.8]	1.7	[1.4–1.8]	1.7	[1.58–1.8]	0.949
BMI, kg/m^2^	26.5	[19.2–36.8]	25.4	[20.0–31.9]	24.7	[20.3–31.8]	0.380
Waist circumference, cm	85.5	[64.0–113.0]	83.0	[70.0–105.0]	84.0	[68.0–101.0]	0.543
Hip circumference, cm	107.0	[90.0–123.0]	104.0	[89.0–115.0]	101.5	[90.0–122.0]	0.391
WHR	0.8	[0.7–1.0]	0.8	[0.7–1.0]	0.8	[0.7–1.0]	0.952
Abdomen circumference, cm	99.5	[69.0–120.0]	98.0	[81.0–110.0]	97.0	[75–115.0]	0.501
Glucose, mg/dL	86.5	[72.0–212.0]	82.0	[72.0–123.0]	79.0	[66.0–88.0]	0.051
Cholesterol, mg/dL ^1^	193.5	[136.0–269]	193.5	[139.0–228.0]	185.0	[145–269.0]	0.486
HDL, mg/dL ^1^	55.5	[31.0–94.0]	58.0	[34.0–79.0]	63.0	[41.0–82.0]	0.176
LDL, mg/dL ^1^	117.0	[67.0–171.0]	111.0	[67.0–157.0]	110.5	[55.0–167.0]	0.535
Triglycerides, mg/dL ^1^	96.0	[44.0–365.0]	90.0	[50.0–267.0]	90.5	[43.0–164.0]	0.740
Cortisol, µg/L ^1^	119.5	[61.0–268.0]	141.5	[85.0–226.0]	121.0	[68.0–254.0]	0.471
Insulin, mU/L ^1^	8.9	[3.0–93.3]	10.0	[5.4–35.0]	8.1	[3.2–27.7]	0.334
Systolic BP, mmHg ^1^	120.0	[100.0–140.0]	118.0	[97.0–155.0]	120.0	[107.0–130.0]	0.984
Diastolic BP, mmHg ^1^	72.5	[60.0–90]	70.0	[55.0–90.0]	70.0	[60.0–90.0]	0.514
MS, *n* (%) ^1^							0.719
No	19	(79.2)	19	(86.4)	11	(91.7)	
Yes	5	(20.8)	3	(13.6)	1	(8.3)	
INF-γ ^1^	0	[0–2.3]	0	[0–2.8]	0.2	[0–7.5]	0.151
IL-6 ^1^	1.0	[0–55.5]	1.6	[0–5.7]	1.3	[0–254.4]	0.489
IL-10 ^1^	0.1	[0–15.0]	0.4	[0–1.9]	0.7	[0–4.5]	0.520
IL-17A ^1^	0	[0–3.6]	0	[0–3.3]	0.5	[0–18.8]	0.411
TNFα ^1^	0	[0–11.0]	0.345	[0–5.3]	0.6	[0–11.9]	

BMI: body mass index; WHR: waist-to-hip ratio; HDL: high-density lipoprotein; LDL: low-density lipoprotein; BP: blood pressure; MS: metabolic syndrome. ^1^ With the exception of smoking habit and MS, missing values were present only for one patient. ^2^ No subjects in our study sample reached the maximum IMI score of 11.

**Table 3:** Factor loading matrix ^1^ and explained variances for the three major dietary patterns identified by principal component factor analysis on baseline nutrient information. Italy, 2018–2019.

Nutrient	Dietary Pattern		
	Animal Products	Vitamins and Fiber	Regional
Animal protein	**0.96**	-	-
Vegetable protein	0.34	0.48	**0.73**
Cholesterol	**0.88**	0.15	0.13
Saturated fatty acids	**0.80**	0.43	0.13
Monounsaturated fatty acids	0.48	**0.66**	0.42
Linoleic acid	**0.64**	0.40	0.44
Linolenic acid	0.49	0.61	0.40
Other polyunsaturated fatty acids	-	-	**0.70**
Soluble carbohydrates	0.43	**0.66**	0.29
Starch	0.46	0.19	**0.70**
Sodium	**0.78**	0.30	0.27
Calcium	**0.66**	0.54	-
Potassium	0.61	**0.72**	0.25
Phosphorus	**0.80**	0.44	0.31
Iron	0.54	0.57	0.57
Zinc	**0.82**	0.29	0.42
Thiamin (vitamin B1)	**0.69**	0.44	0.41
Riboflavin (vitamin B2)	**0.70**	0.43	-
Vitamin B6	**0.73**	0.46	0.32
Total folate	0.33	**0.77**	0.44
Niacin	**0.83**	0.29	0.27
Vitamin C	0.21	**0.88**	-
Retinol	**0.72**	-	0.17
Beta-carotene	-	**0.87**	0.24
Vitamin D	**0.79**	0.10	-
Vitamin E	0.28	**0.76**	0.47
Total fiber	0.22	**0.80**	0.47
**Proportion of explained variance (%)**	37.99	27.28	15.09
**Cumulative explained variance (%)**	37.99	65.27	80.36

^1^ Estimates from a principal component factor analysis on 27 nutrients. For each factor, loadings greater or equal to 0.63 indicated important or “dominant nutrients” in the current paper and were shown in bold typeface; loadings smaller than 0.1 were suppressed.

**Table 4:** Description of the dietary patterns identified at baseline from cluster analysis ^1^: cluster size (i.e., number of subjects included in each cluster) and cluster centers. Italy, 2018–2019.

Cluster Name ^2^	Cluster Size	Cluster Center (Medoid)		
		Animal Products	Vitamins and Fiber	Regional
C1-High consumers	11	0.11	0.24	**0.88** ^3^
C2-Low consumers	19	−0.72	−0.59	−0.41
C3-Omnivorous with meat prevalence	18	**0.81** ^3^	−0.30	−0.30
C4-Omnivorous with plant-based foods prevalence	11	−0.69	**0.70** ^3^	−0.70

^1^ Estimates from the Partitioning Around Medoids clustering algorithm carried out on the factor scores derived from a previous Principal Component Factor Analysis on nutrient information at baseline. The optimal number of clusters was equal to four, as derived from a combination of criteria, including results of the average silhouette method, parsimony and cluster interpretation. ^2^ Cluster names were based on the position of center coordinate within the range of the factor scores used as input data. Specifically, coordinates exceeding the third quartile (in absolute value) indicated extreme dietary behavior. Quartiles (Q) of the factor scores at baseline were as follows: “Animal products” pattern: Q1: −0.69; Q2: −0.24; Q3: 0.48; “Vitamins and fiber” pattern: Q1: −0.53; Q2: −0.19; Q3: 0.37; “Regional”pattern: Q1: −0.60; Q2: −0.30; Q3: 0.32. ^3^ For each cluster, center coordinates greater than or equal to the third quartile score are shown in bold typeface.

## Data Availability

The data presented in this study are available on request from the corresponding author. Sequencing data are accessible at the National Center for Biotechnology Information Sequence Read Archive (NCBI SRA; Bioproject ID PRJNA726866).

## Supplementary Materials

The following are available online at https://www.mdpi.com/article/10.3390/nu13062081/s1, Figure S1: Phylum- and family level composition of the gut microbiota of study subjects compared to healthy Italian controls, Figure S2: Scatter plots of correlations between taxon relative abundances and levels of biochemical parameters (A), cytokines (B) and urinary metabolites (C) in all study subjects, Figure S3: Relationship between the gut microbiota and dietary habits in subjects at risk for metabolic syndrome, Figure S4: PCA score plot from all study participants at baseline, Figure S5: Violin plots for statistically significant changes in systolic blood pressure (within SA-group), cortisol (within PROB-group), and insulin (within PROB-group) between T0 and T30, Figure S6: Impact on the gut microbiota diversity of a diet with fresh foods from organic symbiotic agriculture (SA-group), (A) versus probiotic supplementation (PROB-group), (B), Figure S7: Baseline variation in the relative abundances of genera that varied significantly during intervention within the SA-group, Figure S8: Microbiota compositional variations in SA-group subjects at risk for metabolic syndrome with different long-term dietary habits, Figure S9: Scatter plots and correlation coefficients between taxon relative abundances and levels of anthropometric/biochemical parameters (A) and urinary metabolites (B) in subjects at risk for metabolic syndrome, during the intervention in SA-group., Figure S10: Relationship between changes in genus relative abundances and improvements in metabolic syndrome components over time and within the SA-group, Figure S11: S-Line plot for the OPLS-DA model built for the SA-group (A) and for the OPLS-DA model built for PROB-group (B), Table S1: Factorability of the correlation matrix of the original nutrients: Bartlett’s test of sphericity and measures of sampling adequacy, Table S2: Anthropometric, biochemical, and immunological characteristics for all study participants and separately for the dietary intervention groups at baseline (T0) and after intervention (T30).
