# Supplementary material for: Effects of a Diet Based on Foods from Symbiotic Agriculture on the Gut Microbiota of Subjects at Risk for Metabolic Syndrome
Source: Nutrients. 2021 Jun 17;13(6):2081. doi: 10.3390/nu13062081 (PMC8235411; doi:10.3390/nu13062081)
Supplement: Supplementary file 1 [file nutrients-13-02081-s001.zip › nutrients-1253820-supplementary.pdf]

Supplementary Materials

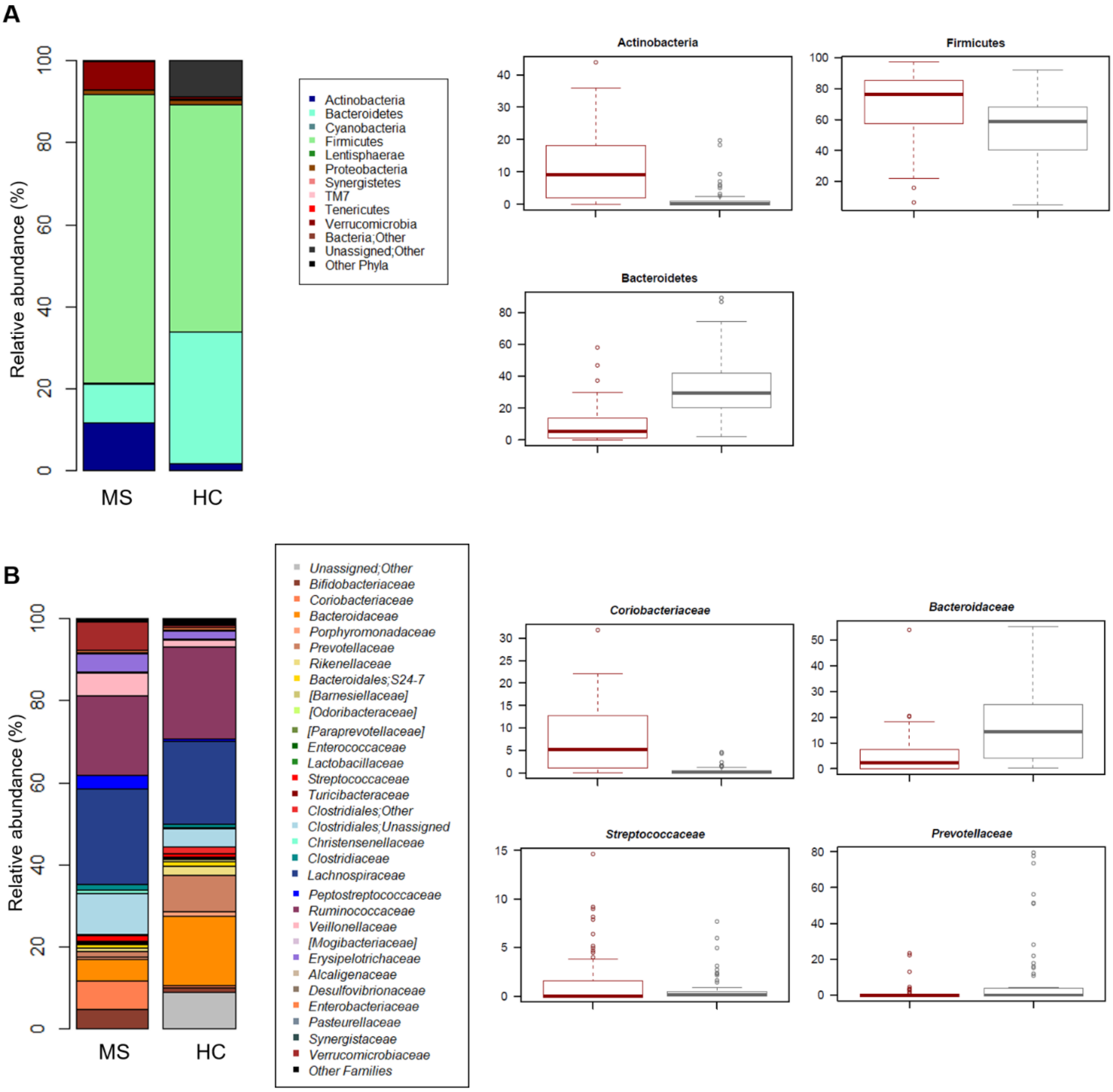

Supplementary Figure S1. Phylum- and family-level composition of the gut microbiota of study subjects compared to healthy Italian controls.

The compositional profiles are shown at the phylum (**A**) and family (**B**) level. For each panel, the bar plots (left) show the major taxa and the boxplots (right) the relative abundance distribution of differentially represented taxa between study subjects and healthy Italian controls ($p \leq 0.05$; Wilcoxon test). Only taxa with relative abundance >0.1% in at least 2 samples are shown. Subjects at risk for metabolic syndrome are indicated with MS on the left and colored in dark red on the right; healthy controls are indicated with HC and colored in grey.

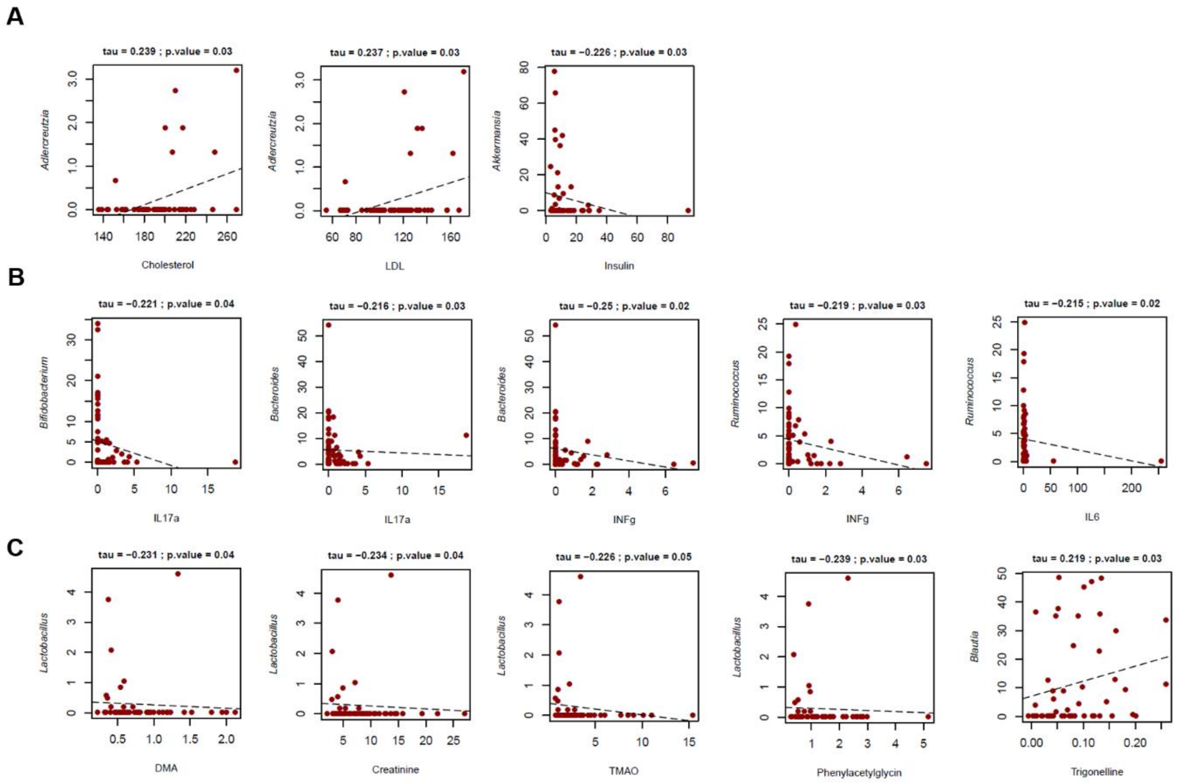

Supplementary Figure S2. Scatter plots of correlations between taxon relative abundances and levels of biochemical parameters (A), cytokines (B) and urinary metabolites (C) in all study subjects.

Only statistically significant correlations ($p \leq 0.05$) based on Kendall rank correlation test are shown.

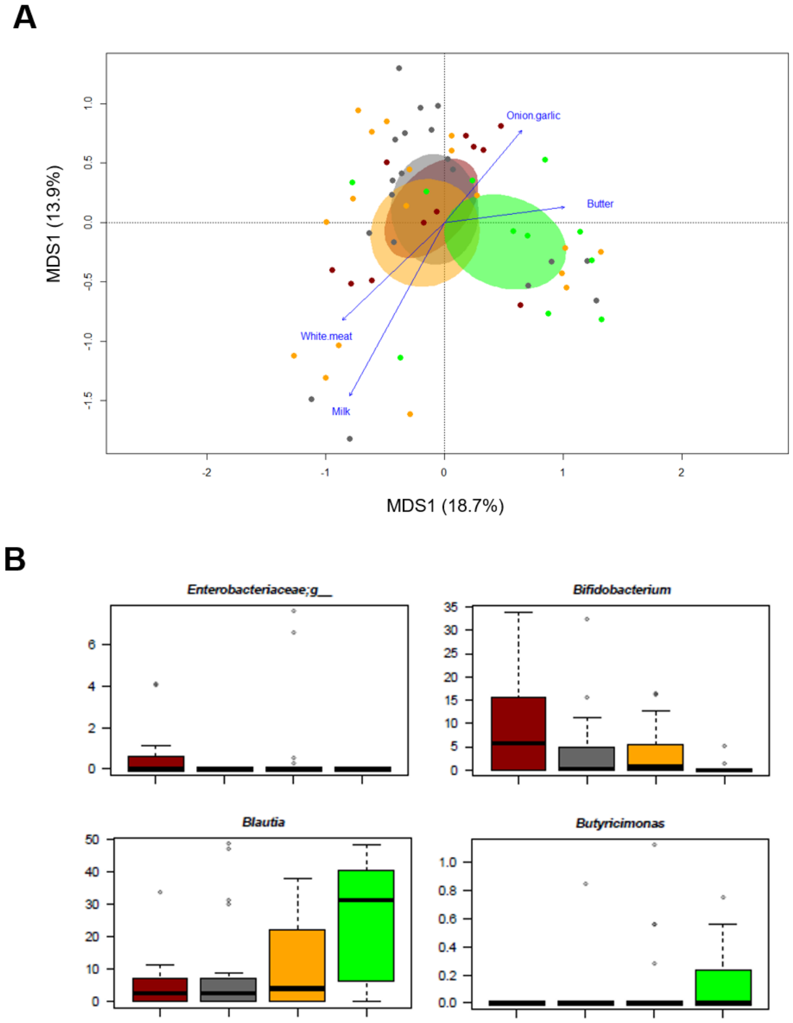

Supplementary Figure S3. Relationship between the gut microbiota and dietary habits in subjects at risk for metabolic syndrome.

(**A**) PCoA plot of inter-sample diversity, based on Bray–Curtis distances between the genus-level profiles of subjects with different dietary patterns. Red, “High consumers” individuals; grey, “Low consumers” individuals; orange, “Omnivorous with meat prevalence” individuals; green, “Omnivorous with plant-based foods prevalence” individuals. See also Table 4. A significant separation was found between the “Omnivorous with plant-based foods prevalence”-related microbiota and the others (p=0.05, permutation test with pseudo-F ratio). Food groups and condiments with the largest contribution to the ordination space are indicated with blue arrows (p≤0.1, permutational correlation test, “envfit” function). (**B**) Boxplots showing the relative abundance distribution of differentially represented genera among groups (p≤0.1, Kruskal-Wallis test). Same color code as in A.

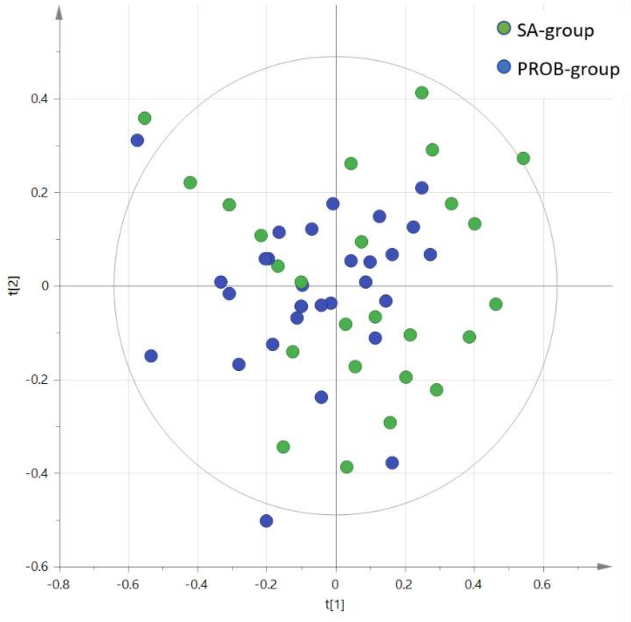

Supplementary Figure S4. PCA score plot from all study participants at baseline.

Data points for subjects in SA-group are represented in green whereas those in PROB-group in blue. The outer ellipse represents the 95% confidence interval (Hotelling's T2).

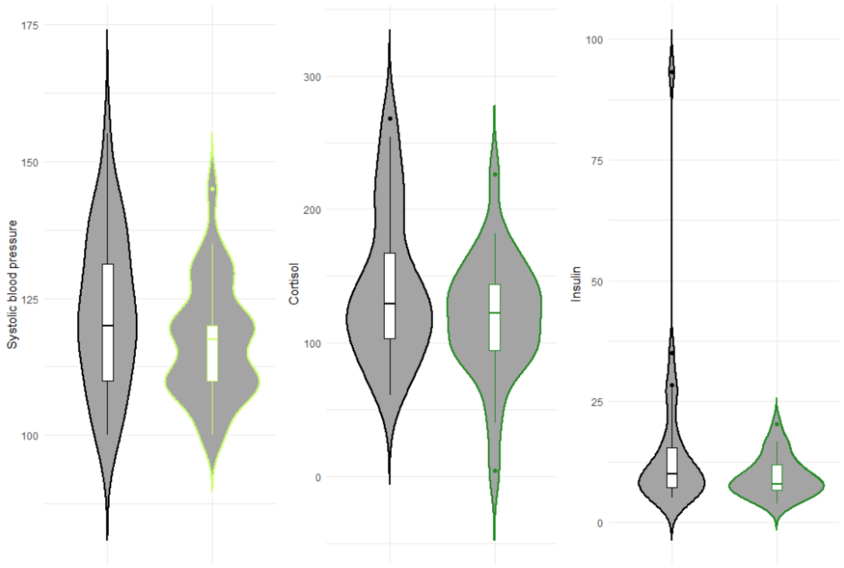

Supplementary Figure S5. Violin plots for statistically significant changes in systolic blood pressure (within SA-group), cortisol (within PROB-group), and insulin (within PROB-group) between T0 and T30.

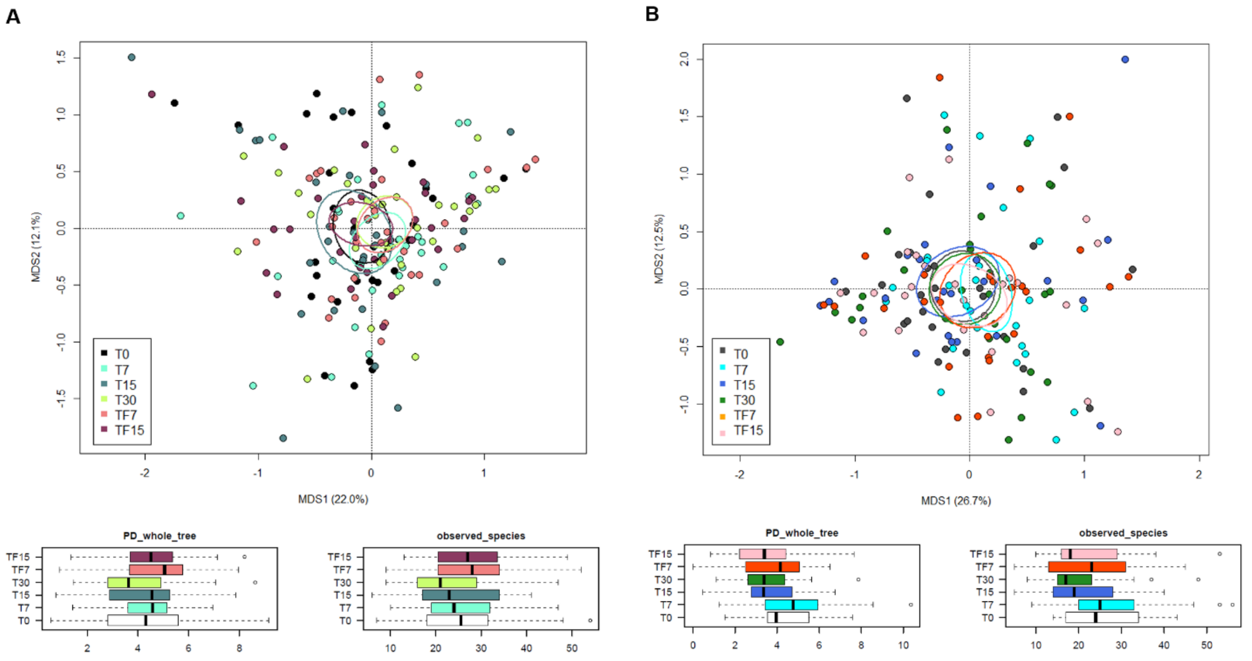

Supplementary Figure S6. Impact on the gut microbiota diversity of a diet with fresh foods from organic symbiotic agriculture (SA-group, A) versus probiotic supplementation (PROB-group, B).

For each panel, top, PCoA plot of inter-sample diversity, based on weighted UniFrac distances between the microbiota profiles of subjects at risk for metabolic syndrome during the intervention period; bottom, boxplots showing the distribution of alpha diversity over time, according to Faith's Phylogenetic Diversity (PD_whole_tree) and the number of observed ASVs. A temporal reduction in alpha diversity was observed in PROB-group, with the lowest values after 30 days of intervention ($p \leq 0.04$, Friedman test). The microbiota diversity was assessed at baseline (T0), after 7 (T7), 15 (T15) and 30 (T30) days of intervention, and at follow-up, 7 (TF7) and 15 (TF15) days after the end of the intervention.

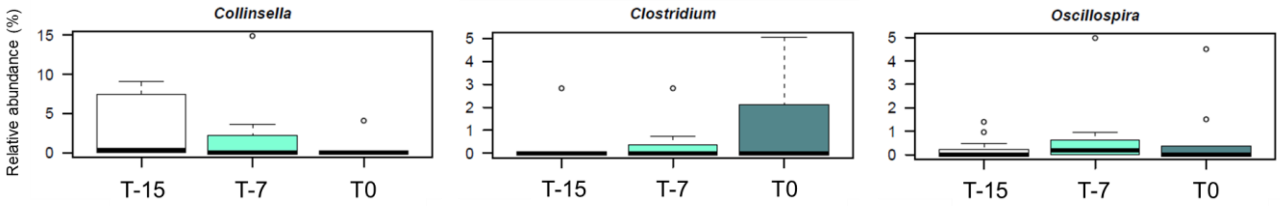

Supplementary Figure S7. Baseline variation in the relative abundances of genera that varied significantly during intervention within the SA-group. Boxplots showing the relative abundance distribution of *Collinsella*, *Clostridium* and *Oscillospira* in subjects at risk for metabolic syndrome in the 2 weeks prior to SA-based diet. No differences were found ($p>0.05$, Friedman test).

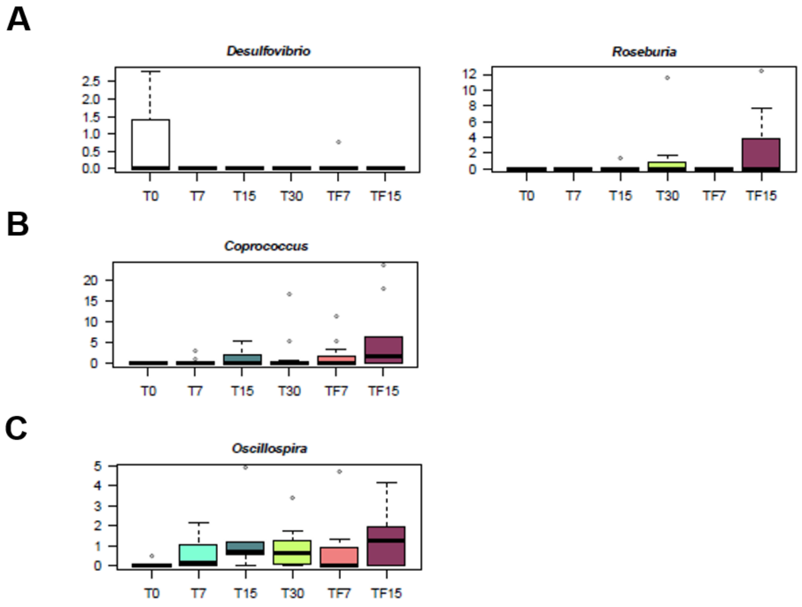

Supplementary Figure S8. Microbiota compositional variations in SA-group subjects at risk for metabolic syndrome with different long-term dietary habits.

Boxplots showing the relative abundance distribution of differentially represented genera over time in "High consumers" (**A**), "Low consumers" (**B**), and "Omnivorous with meat prevalence" (**C**) clusters ($p \leq 0.1$, Friedman test). For subject stratification based on cluster analysis, see also Table 4.

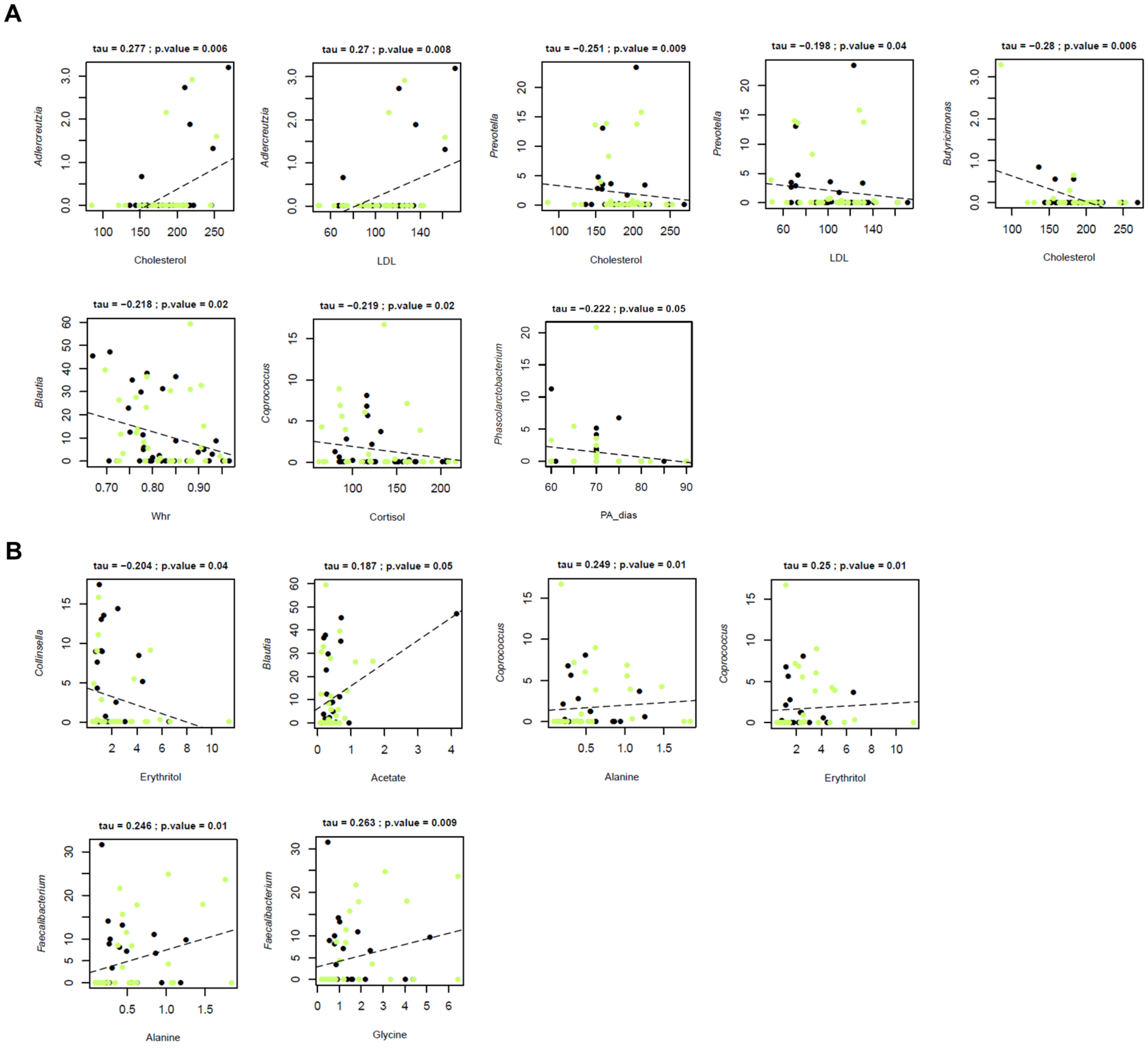

Supplementary Figure S9. Scatter plots and correlation coefficients between taxon relative abundances and levels of anthropometric/biochemical parameters (A) and urinary metabolites (B) in subjects at risk for metabolic syndrome, during the intervention in SA-group.

Samples collected at baseline and after 30 days of intervention are identified with black and green dots, respectively. Only statistically significant correlations ($p \leq 0.05$) based on Kendall rank correlation test are shown. Whr, waist-hip ratio; PA_dias, diastolic blood pressure.

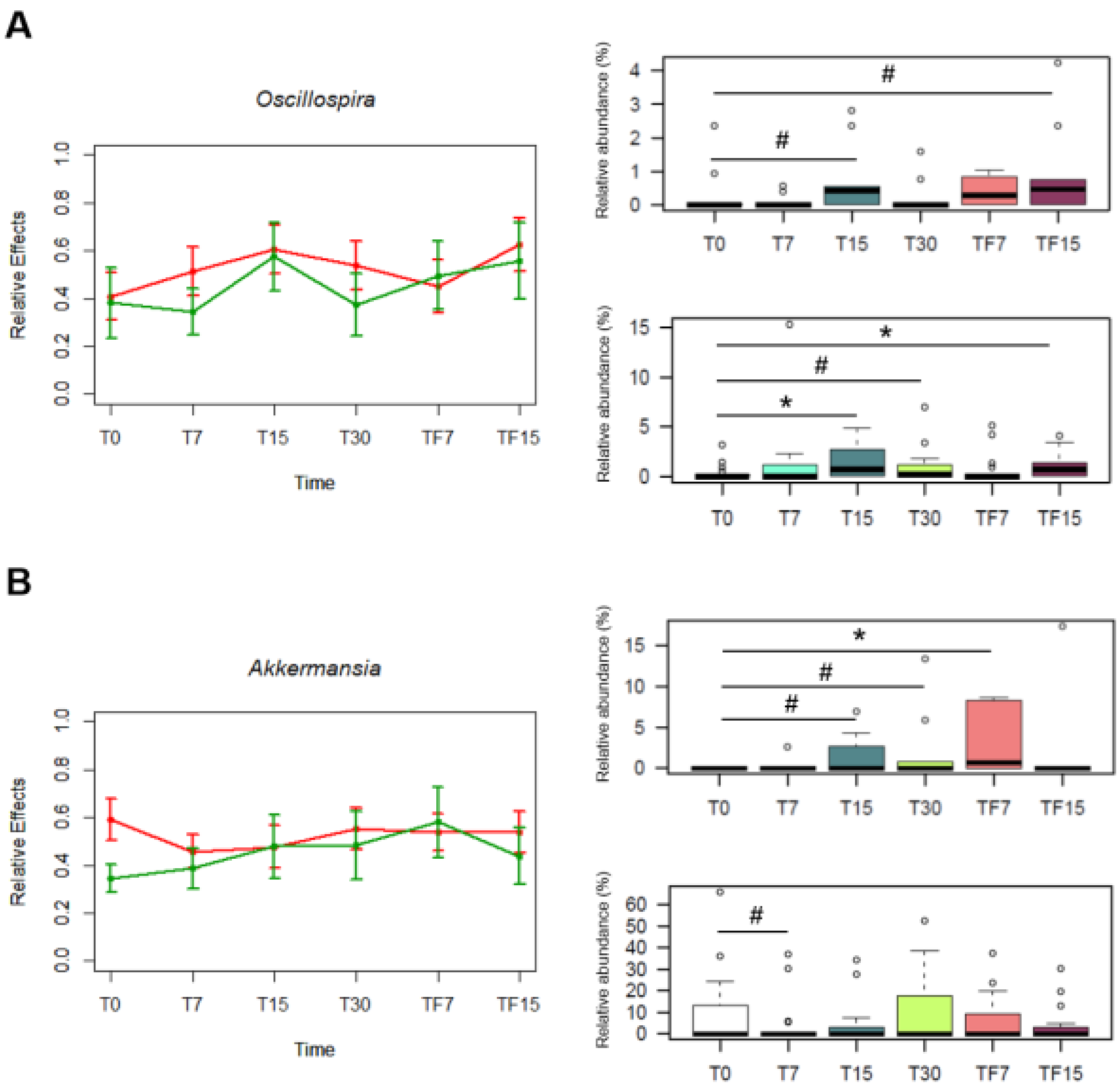

Supplementary Figure S10. Relationship between changes in genus relative abundances and improvements in metabolic syndrome components over time and within the SA-group.

A nonparametric rank-based longitudinal analysis led to the identification of two genera, i.e., *Oscillospira* (**A**) and *Akkermansia* (**B**), of which the first increased over time in a similar way in both subjects who experienced improvement in at least one risk factor for metabolic syndrome and in those who worsened or did not change, and the latter showed a different trend between groups. For each panel, left, relative effects with 95% confidence interval for the two subject groups (green, improved; red, stationary/worsened) (A, p=0.01; B, p=0.08; ANOVA-type statistics); right, boxplots showing the relative abundance distribution of each taxon in subjects who improved (top) vs those who worsened or did not change (bottom) (*, p≤0.05; #, 0.05<p≤0.1; Wilcoxon test). The gut microbiota was profiled at baseline (T0), after 7 (T7), 15 (T15) and 30 (T30) days of intervention, and at follow-up, 7 (TF7) and 15 (TF15) days after the end of the intervention.

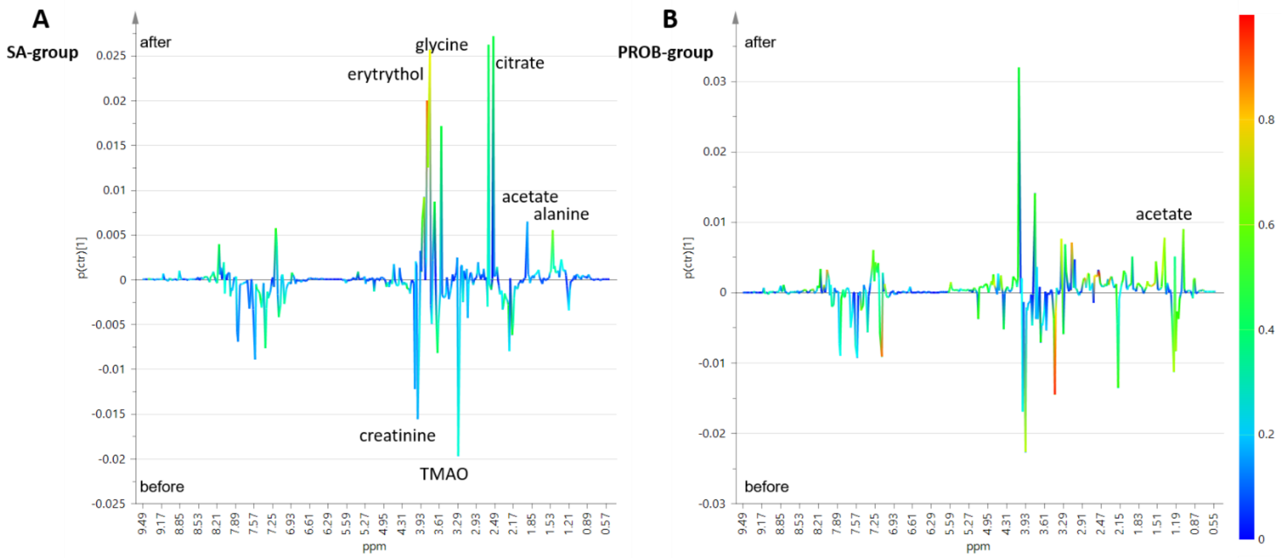

Supplementary Figure S11. S-Line plot for the OPLS-DA model built for the SA-group (A) and for the OPLS-DA model built for PROB-group (B).

Supplementary Table S1. Factorability of the correlation matrix of the original nutrients: Bartlett's test of sphericity and measures of sampling adequacy.

**Bartlett's test of sphericity:** p-value< 0.001	
**Overall measure of sampling adequacy (Kaiser-Meyer-Olkin statistic)** [1]**:** 0.84	
**Individual measures of sampling adequacy:**	
0.60-0.69	other polyunsaturated fatty acids, calcium
0.70-0.79	animal protein, saturated fatty acids, soluble carbohydrates, starch, riboflavin
0.80-0.89	cholesterol, thiamin, vitamin D, vitamin E, folate, vegetable protein, monounsaturated fatty acids, potassium, vitamin C, phosphorus, niacin
≥0.90	zinc, vitamin B6, beta-carotene, total fiber, retinol, linolenic acid, iron, linoleic acid, sodium

[1] Overall and individual measures of sampling adequacy range between 0 and 1, with values > 0.60 indicating a satisfactory size.

Supplementary Table S2. Anthropometric, biochemical, and immunological characteristics for all study participants and separately for the dietary intervention groups at baseline (T0) and after intervention (T30).

	All			SA-group			PROB-group		
	**T0**	**T30**	**P**	**T0**	**T30**	**P**	**T0**	**T30**	**P**
	median [min - max]	median [min - max]		median [min - max]	median [min - max]		median [min - max]	median [min - max]	
**Weight, kg**	70.5 [44.0 – 103.0]	69.5 [44.0 – 105.0]	0.120	70.0 [44.0 – 103.0]	68.0 [44.0 – 105.0]	**0.081**	72.0 [47.0 – 94.5]	70.5 [45.0 – 96.0]	0.782
**BMI, kg/m2**	25.7 [19.2 – 36.8]	25.4 [19.0 – 36.8]	**0.088**	26.1 [19.2 – 36.8]	25.4 [19.0 – 36.8]	**0.057**	25.3 [19.8 – 33.3]	25.3 [19.0 – 33.5]	0.724
**Waist circumference, cm**	85.0 [64.0 – 113.0]	84.5 [61.0 – 112.0]	0.935	85.0 [67.0 – 113.0]	85.0 [68.0 – 112.0]	0.857	84.0 [64.0 – 102.0]	84.0 [61.0 – 100.0]	0.724
**Hip circumference, cm**	105.0 [89.0 – 123.0]	104.5 [87.0 – 123.0]	0.215	105.0 [89.0 – 123.0]	105.0 [90.0 – 123.0]	0.148	104.0 [90.0 – 116.0]	104.0 [87.0 – 117.0]	0.887
**WHR**	0.8 [0.7 – 1.0]	0.8 [0.7 – 1.0]	0.478	0.8 [0.7 – 1.0]	0.8 [0.7 – 1.0]	0.302	0.8 [0.7 – 1.0]	0.8 [0.7 – 0.9]	0.755
**Abdomen circumference, cm**	98.5 [69.0 – 120.0]	98.0 [64.0 – 119.0]	0.769	98.0 [78.0 – 120.0]	97.0 [79.0 – 119.0]	0.809	99.0 [69.0 – 111.0]	98.0 [64.0 – 111.0]	0.717
**Glucose, mg/dl**	82.5 [66.0 – 212.0]	83.0 [62.0 – 186.0]	0.141	83.0 [66.0 – 212.0]	83.0 [66.0 – 186.0]	**0.067**	82.0 [72.0 – 103.0]	83.0 [62.0 – 116.0]	0.597
**Cholesterol, mg/dl**[1]	193.0 [136.0 – 269.0]	189.0 [85.0 – 253.0]	0.104	190.0 [136.0 – 269.0]	185.5 [85.0 – 253.0]	0.184	195.0 [139.0 – 269.0]	194.0 [150.0 – 249.0]	0.368
**HDL, mg/dl**[1]	59.0 [31.0 – 94.0]	59.0 [33.0 – 85.0]	0.791	65.0 [31.0 – 94.0]	63.0 [33.0 – 85.0]	0.438	55.0 [34.0 – 86.0]	52.0 [33.0 – 76.0]	0.330
**LDL, mg/dl**[1]	113.0 [55.0 – 171.0]	111.0 [49.0 – 171.0]	0.367	106.0 [67.0 – 171.0]	109.2 [49.0 162.0]	0.486	119.0 [55.0 – 167.0]	116.0 [70.0 – 171.0]	0.580
**Triglycerides, mg/dl**[1]	90.0 [43.0 – 365.0]	93.0 [45.0 – 307.0]	0.265	91.5 [43.0 – 365.0]	87.5 [49.0 – 307.0]	0.144	90.0 [44.0 – 243.0]	101.4 [45.0 – 300.0]	0.968
**Cortisol, μg/l**[1]	125.0 [61.0 – 268.0]	121.0 [4.0 – 226.0]	**0.071**	124.5 [68.0 – 206.0]	120.5 [62.0 – 217.0]	0.739	129.0 [61.0 - 268]	122.0 [4.0 – 226.0]	**0.020**
**Insulin, mU/L**[1]	9.2 [3.0 – 93.3]	7.9 [3.1 – 35.6]	**0.013**	8.9 [3.0 – 28.2]	7.7 [3.1 – 35.6]	0.624	10.0 [5.1 – 93.3]	7.9 [3.9 – 20.3]	**0.006**
**Systolic BP, mmHg**[1]	120.0 [97.0 – 155.0]	115.0 [100.0 – 140.0]	**0.056**	120.0 [100.0 – 155.0]	115.0 [100.0 – 135.0]	**0.032**	119.5 [97.0 – 150.0]	117.9 [100.0 – 140.0]	0.858
**Diastolic BP, mmHg**[1]	71.5 [55.0 – 90.0]	70.0 [60.0 – 90.0]	**0.097**	70.0 [60.0 – 90.0]	70.0 [60.0 – 90.0]	0.218	75.0 [55.0 – 90.0]	70.0 [60.0 – 86.0]	0.271
**MS, n (%)**[1]			**<0.001**			**0.001**			**0.002**
No	40 (81.6)	43 (87.8)		23 (79.3)	25 (86.2)		17 (85.0)	18 (90.0)	
Yes	9 (18.4)	6 (12.2)		6 (20.7)	4 (13.8)		3 (15.0)	2 (10.0)	
**INF-γ**[1]	0 [0.0 – 7.5]	0 [0.0 – 13.43]	0.216	0 [0.0 -2.8]	0 [0.0 – 13.4]	0.379	0 [0.0 – 7.5]	0 [0.0 – 4.3]	0.359
**IL-6**[1]	1.3 [0.0 – 254.4]	1.5 [0.0 – 537.8]	0.962	1.3 [0.0 – 55.5]	0.7 [0.0 – 42.6]	0.100	1.6 [0.0 – 254.4]	1.7 [0.0 – 537.8]	0.162
**IL-10**[1]	0.3 [0.0 – 15.0]	0.4 [0.0 – 23.8]	0.980	0 [0.0 – 15.0]	0.1 [0.0 – 23.8]	0.644	0.6 [0.0 – 4.5]	0.8 [0.0 – 4.3]	0.681
**IL-17A**[1]	0 [0.0 – 18.8]	0 [0.0 – 18.5]	0.228	0 [0.0 – 2.6]	0 [0.0 – 18.5]	1.000	0.8 [0.0 – 18.8]	0 [0.0 – 9.1]	0.126
**TNFα**[1]	0.2 [0.0 – 67.9]	0 [0.0 – 40.8]	0.830	0 [0.0 – 67.9]	0.5 [0.0 – 40.8]	0.225	0.3 [0.0 – 11.9]	0 [0.0 – 2.1]	0.237

BMI: body mass index; WHR: waist-to-hip ratio; HDL: high-density lipoprotein; LDL: low-density lipoprotein; BP: blood pressure; MS: metabolic syndrome.

[1] Missing values were generally present for one subject only, with the exception of systolic, diastolic blood pressure, and MS, for which there were 11 missing values.
